# Cross‐tissue multi‐omics analyses reveal the gut microbiota's absence impacts organ morphology, immune homeostasis, bile acid and lipid metabolism

**DOI:** 10.1002/imt2.272

**Published:** 2025-02-14

**Authors:** Juan Shen, Weiming Liang, Ruizhen Zhao, Yang Chen, Yanmin Liu, Wei Cheng, Tailiang Chai, Yin Zhang, Silian Chen, Jiazhe Liu, Xueting Chen, Yusheng Deng, Zhao Zhang, Yufen Huang, Huanjie Yang, Li Pang, Qinwei Qiu, Haohao Deng, Shanshan Pan, Linying Wang, Jingjing Ye, Wen Luo, Xuanting Jiang, Xiao Huang, Wanshun Li, Elaine Lai‐Han Leung, Lu Zhang, Li Huang, Zhimin Yang, Rouxi Chen, Junpu Mei, Zhen Yue, Hong Wei, Kristiansen Karsten, Lijuan Han, Xiaodong Fang

**Affiliations:** ^1^ BGI Research Shenzhen China; ^2^ Qingdao‐Europe Advanced Institute for Life Sciences BGI Research Qingdao China; ^3^ State Key Laboratory of Traditional Chinese Medicine Syndrome, State Key Laboratory of Dampness Syndrome of Chinese Medicine Syndrome The Second Affiliated Hospital of Guangzhou University of Chinese Medicine Guangzhou China; ^4^ College of Animal Sciences and Technology Huazhong Agricultural University Wuhan China; ^5^ BGI Research Beijing China; ^6^ BGI Research Qingdao China; ^7^ Kangmeihuada (KMHD) GeneTech Co., Ltd. Shenzhen China; ^8^ Zhuhai UM Science & Technology Research Institute‐Kangmeihuada (KMHD) joint lab Zhuhai China; ^9^ Cancer Center, Faculty of Health Sciences University of Macau Macau (SAR) China; ^10^ MOE Frontiers Science Center for Precision Oncology University of Macau Macau (SAR) China; ^11^ Department of Computer Science Hong Kong Baptist University Hong Kong China; ^12^ BGI Research Sanya China; ^13^ Yu‐Yue Pathology Scientific Research Center Chongqing China; ^14^ Laboratory of Genomics and Molecular Biomedicine, Department of Biology University of Copenhagen Copenhagen Denmark; ^15^ Kangmei Pharmaceutical Co., Ltd. Jieyang China

**Keywords:** aggregation index, bile acid and lipid metabolism, germ‐free mice, immune homeostasis, lipid droplet, spatial transcriptomics and single‐cell RNA sequencing, zonation

## Abstract

The gut microbiota influences host immunity and metabolism, and changes in its composition and function have been implicated in several non‐communicable diseases. Here, comparing germ‐free (GF) and specific pathogen‐free (SPF) mice using spatial transcriptomics, single‐cell RNA sequencing, and targeted bile acid metabolomics across multiple organs, we systematically assessed how the gut microbiota's absence affected organ morphology, immune homeostasis, bile acid, and lipid metabolism. Through integrated analysis, we detect marked aberration in B, myeloid, and T/natural killer cells, altered mucosal zonation and nutrient uptake, and significant shifts in bile acid profiles in feces, liver, and circulation, with the alternate synthesis pathway predominant in GF mice and pronounced changes in bile acid enterohepatic circulation. Particularly, autophagy‐driven lipid droplet breakdown in ileum epithelium and the liver's zinc finger and BTB domain‐containing protein (ZBTB20)‐Lipoprotein lipase (LPL) (ZBTB20‐LPL) axis are key to plasma lipid homeostasis in GF mice. Our results unveil the complexity of microbiota–host interactions in the crosstalk between commensal gut bacteria and the host.

## Highlights

- Single‐cell, spatial transcriptomics, and bile acidomics atlases in germ‐free mice.
- Marked aberration and tissue heterogeneity in B, myeloid, and T/NK cells in germ‐free mice.
- Microbiota shapes mucosal zonation and modulates lipid dynamics of the small intestine.
- Germ‐free mice show liver bile acid synthesis and ileal reabsorption anomalies.

## Graphical abstract

We present a multi‐organ single‐cell, spatial transcriptomics, and BA omics atlas of specific pathogen‐free (SPF) and germ‐free (GF) mice. We found plasma cell aggregation displays significant tissue heterogeneity depending on the gut microbiota. GF mice exhibit impaired follicular and marginal zone B cell maturation, linked to microbiota‐mediated modulation of *Cr2* gene expression. The microbiota regulates the development and survival of neutrophils in the bone marrow, influences the development and differentiation of T cells in the thymus, and modulates intraepithelial γδ T cell composition and lipid absorption in the small intestine. The absence of microbiota in GF mice alters the intestinal mucosa zonation and triggers coordinated dynamics in intestinal lipid absorption, transport, chylomicron synthesis, lipid droplet formation, lipolysis, and fatty acid oxidation in the small intestine enterocytes. The liver microbiota‐dependent zinc finger and BTB domain‐containing protein (ZBTB20)‐Lipoprotein lipase (LPL) axis plays a role in plasma lipid homeostasis.

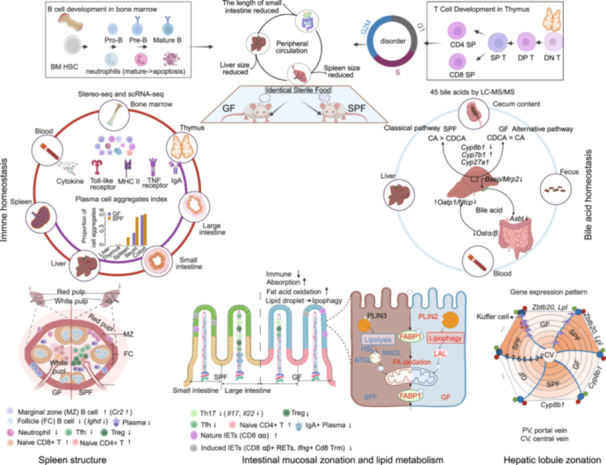

## INTRODUCTION

The gastrointestinal tract hosts diverse communities of commensal bacteria [1], which exhibit characteristic compartmentalization profoundly influencing many aspects of host physiology [2], including organizational structures [3, 4], immunity [5, 6], circadian rhythm [7, 8], nutritional responses [6, 9], energy, and lipid metabolism [5, 10, 11, 12, 13]. Germ‐free (GF) animals, mostly mice, have become a widely used tool for examining the impact of the microbiota on host physiology. Research using GF mouse models has revealed that the development and morphogenesis of numbers of host organs, including the gastrointestinal tract [3], spleen [14], lymph nodes [3], liver [15], skeletal muscle [16], gallbladder [17], and fat [10], are regulated by the gut microbiota. However, most of these findings still concern the anatomical level and biochemical observation, lacking integrated research at the cellular, molecular, and tissue spatial transcritomic level.

The interactions between the gut microbiota and host immunity are dynamic, diverse, and environment‐dependent across tissues [18]. Recent studies have revealed that many immune organs crosstalk with microbes [4, 19, 20, 21]. In addition, the microbiota significantly influences glucose and lipid metabolism, playing a key role in maintaining energy homeostasis in part via the hepatoenteric axis [19, 20]. Although pioneering research has established a robust groundwork for comprehending the interplay between the microbiota and the host immune system, there is a scarcity of investigations into the pattern of immune cells across the entire host organism, particularly in terms of spatial distribution characteristics, the microenvironment at a finer structural level, and the possible crosstalk of immune effector molecules among different organs. Finally, a comprehensive understanding of the underlying mechanisms by which the microbiota–gut–liver axis affects host lipid metabolism and functional zonation in the intestinal epithelium and hepatic lobules is still lacking.

In this study, single‐cell RNA sequencing (scRNA‐seq), spatial enhanced resolution omics‐sequencing (Stereo‐seq), and targeted analysis of bile acid (BA) metabolism were used to obtain multi‐omics data from different tissues and organs comparing GF and specific pathogen‐free (SPF) mice. This design aims to further elucidate how the absence of microbiota disturbs organ morphology, cell composition and function, key cytokines expression, enterohepatic axis circulation of BA, and lipid metabolism. Thus, our study provides a valuable reference map for exploring crosstalk between microbiota and host.

## RESULTS

### Integrated cross‐organ multi‐omics maps of tissues in SPF and GF mice

**Figure 1:**
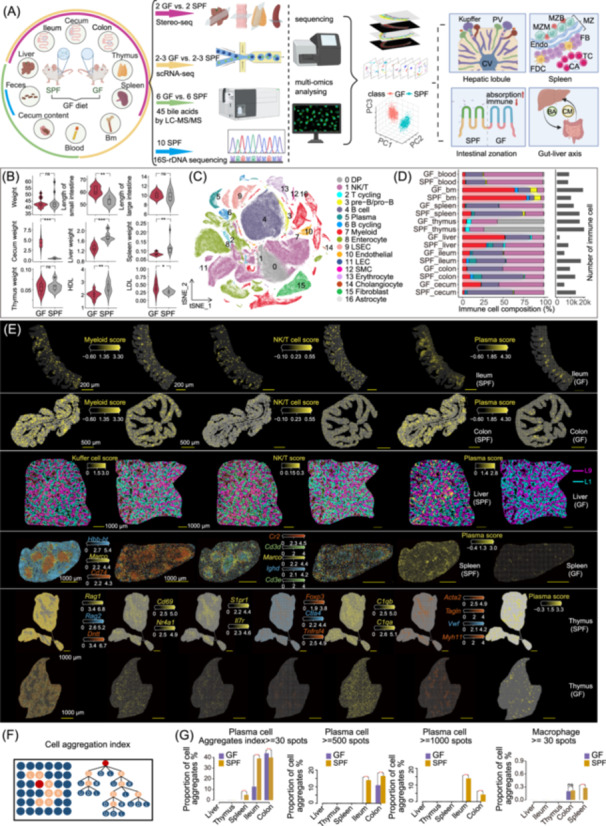
An overview of the multi‐omics study design and the summary of single‐cell RNA sequencing (scRNA‐seq) and spatial enhanced resolution omics‐sequencing (Stereo‐seq) data. (A) Multi‐omics atlas reveals disruptions in tissue microenvironments, immune, and metabolic homeostasis in microbiota‐deficient conditions. (B) Summary of significant differential phenotypes. (C) The t‐Distributed Stochastic Neighbor Embedding (t‐SNE) visualization of clusters colored by major cell types. Legend on the right provides cell type annotations. DP, double positive T cell; NK, natural killer; LEC, lymphatic endothelial cell; SMC, smooth muscle cells; LSEC, liver sinusoidal endothelial cells; pre‐B, precursors of B lymphocytes; pro‐B, progenitor of B lymphocytes. (D) Bar plots showing the percentage and count of immune cells for each tissue after quality control. (E) Visualization depicting the spatial distribution of cells and key genes expression, reflecting distinct physiological function structures of the organ. For the thymus, we primarily utilized crucial marker genes to depict the spatially continuous developmental processes of T cells from the cortex to the medulla, involving the double‐positive (DP) stage (*Dntt*, *Rag1* and *Rag2*), single‐positive (SP) stage (*Nr4a1* and *Cd69*), and maturation stage (*Il7r*, *S1pr1*, *Foxp3*, *Ctla4*, and *Tnfrsf4*) [22, 23]. For the spleen, the marker genes of red pulp (*Hbb‐bt*), white pulp (*Cd74*), and marginal macrophages (*Marco*), as well as the marker genes of fine structure in white pulp including marginal B cells (*Cr2*), follicular B cells (*Ighd*), and T‐cell (*Cd3d* and *Cd3e*) were selected to demonstrate its structure. Spatial visualization of the liver zonation layer 1 (pericentral, CV, L1) and layer 9 (periportal, PV, L9) area. (F) Diagram of cell aggregation index calculated by Breadth‐First‐Search (BFS). (G) The proportion of aggregation index with spots above 30 or above 500 was determined. The Fisher's exact test based on a two‐dimensional contingency table is used to detect the significant differences in the aggregation index between GF and SPF mice (ns *p* > 0.05, *p < 0.05, **p < 0.01, ***p < 0.001 ****p < 0.0001).

In this study, we performed a comprehensive analysis of cross‐organ multi‐omics data (Figure 1A). Both GF and SPF mice were fed an identical, low‐crude fiber (1%–4%) diet sterilized by 50 kGy Co60‐g irradiation for 7 weeks post‐weaning at 3 weeks of age (Table S1). We obtained 16S rRNA sequencing data from ten 10‐week‐old male SPF mice, in which *Muribaculaceae bacterium* DSM 103720, *Bacteroides caecimuris*, *Bacteroides vulgatus*, and *Muribaculum intestinale* collectively account for 35%−90% of the sample (Figure 1A and S1). Through a systematic investigation including eleven 10‐week‐old GF male mice and 10‐week‐old male SPF mice, we observed abnormal white blood cell levels, altered lipoprotein levels, and notable anatomical changes in the length of the small intestine, weight of the cecum, liver, and spleen in GF mice compared to SPF mice (Figure 1B and Table S2). These observations highlighted that the absence of microbiota led to dysregulation of cells involvement in the immune system, lipid homeostasis, organ development, and the cellular microenvironment.

Given these differences, eight tissues including primary (bone marrow and thymus) and secondary (spleen) lymphoid organs, mucosal tissues (gut), as well as blood and liver were used for scRNA‐seq. After filtering, a total of 269,105 cells were retained for unsupervised clustering, with the minimal number of cells being 9281 from the liver of GF mice and the maximum number of cells being 24,170 from the cecum also from GF mice (Figure S2A,B and Table S3). On average, we detected 1457 genes and 5785 unique molecular identifiers (UMIs) per cell (Figure S2C). Unbiased clustering and marker‐gene analysis of the integrated data identified 17 major cell populations (Figures 1C and S3). Reflecting the tissues sampled, we observed a high proportion of immune cells and epithelial cells, accounting for approximately 85.42% of the cell composition (22.89%, 33.83%, 12.32%, and 16.37% for B, NK/T, myeloid, and epithelial cells, respectively) (Figure S3). Single cell analyses based on multi‐tissue integration revealed a large variation in the abundance of different immune cells across tissues (Figure 1D).

To explore the effects of the absence of microbiota on organ structure and immunological niches, six immune‐related tissues were used for Stereo‐seq. After controlling for data quality, a total of 1,085,138 bins of spatial transcriptomics datasets were used for the analysis (Table S3). A fine structure of organs and the spatial localization of different cell types were demonstrated by a series of informative marker genes or gene set scores (Figures 1E, S4 and Table S4). We observed a notable reduction in the proportion of plasma cells across liver, spleen, and gut of GF mice (Figure 1E). However, the proportion of plasma cells in the GF thymus exhibited minimal changes (Figure 1E), consistent with previous studies suggesting that thymic Ig class switching and secretion do not appear to be driven by external antigen stimulation or microbial colonization [21, 24, 25, 26]. To quantify the spatial distribution characteristics of immune cells, we defined an aggregation index based on the connectivity among spots (Figure 1F; Details are shown in Methods). We found that plasma cells tend to form dense clusters, particularly in the intestinal tract, where larger aggregates were observed, constituting 15% of the population in the colon and ileum (Figure 1E,G). This implies that microbes and their products regulate the aggregation behavior of plasma cells. In GF mice colons, plasma cell clusters are less prevalent and smaller in scale compared to those in SPF mice, suggesting weaker food‐derived plasma cell aggregation versus microbial stimulation (Figure 1G). For macrophages, we observed a reduced aggregation index in GF mice spleen, suggesting weaker connectivity and potential immaturity or disorder in their marginal zones marked by *Macro* gene (Figure 1E,G). And, in line with previous studies, liver spatial zonation shows microbiota‐mediated enrichment of Kupffer cells around the portal vein, with GF mice exhibiting mild enrichment [27, 28] (Figure S4A,B). Regarding the expression of genes at the apical layer of intestinal epithelium, such as *Saa1* in the large intestine, there is a trend of increased expression in GF mice, suggesting the impact of microbes on epithelial function (Figure S4C).

Following these spatial findings, we will next focus on microbiota regulation on immune cell maturation and intestinal epithelial zonation.

### The microbiota exhibits organ‐specific heterogeneity in the regulation of B cell development and plasma cell composition

**Figure 2:**
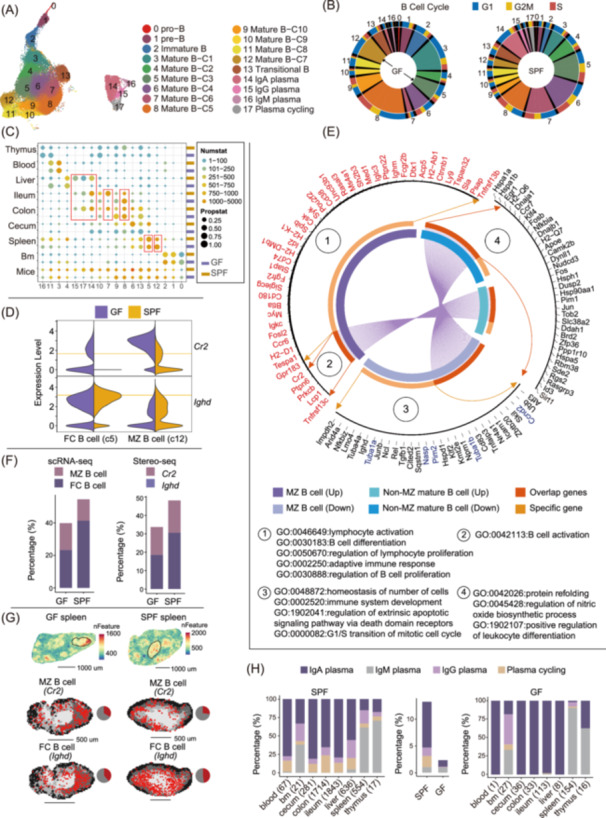
Microbiota‐regulated heterogeneity of the B cell compartment across tissues. (A) The Uniform Manifold Approximation and Projection (UMAP) visualizations of B cell compartment. (B) The proportion and cell cycle of B cell subtypes. The outer circle is the proportion of cells in each phase of the cell cycle for each cluster, and the inner circle is the cell proportion. (C) Heatmaps illustrating the subtype distribution of B cells across each tissue and all tissues between GF and SPF mice. The color gradient represents the range of cell counts in each tissue. Each circle's size indicates the normalized proportion of subtypes, calculated by (*R*‐min(*R*))/(max(*R*)‐min(*R*)), where *R* is the actual subtype proportion of each tissue. (D) The expression of marker genes *Cr2* and *Ighd* in marginal zone (MZ) and follicular (FC) B cells. (E) The differential gene of Gene Ontology (GO) enrichment results of MZ B cells and non‐MZ B cells. The inner circle represents the commonalities and specificities of up‐ and down‐regulated differential genes between MZ B and non‐MZ mature B cells. The outer circle displays key genes enriched in the pathways. (F) The cellular composition of MZ B cells and FC B cells was assessed at the level of scRNA‐seq and Stereo‐seq. (G) Spatial visualization of core genes in the white pulp region of the spleen. (H) Heterogeneous distribution of plasma cell subtypes composition across tissues between GF and SPF. The numbers in parentheses represent the count of plasma cells.

We identified different developmental stages of B cells, encompassing progenitors, immature cells, naive cells, activated cells, and plasma cells, based on the expression of *Vpreb1, Igll1, Dntt, Vpreb3, Rag1, Sox4, Cd24a, Ms4a1, Ighd, Cr2, Hspa1a, Hspa1b, Hsph1, Jchain, Igha, Ighg2b, Ighg2c*, and *Ighm* (Figures 2A and S5A). These stages were distinguished by their distribution patterns between bone marrow and peripheral tissues (Figure S5B). Cell cycle analysis showed a significant perturbation of B cells in the absence of microbiota, especially in subtypes 5 and 12, indicating a potential influence of the microbiota on the spleen (Figures 2B and S5B). A distinct subtype 12 expressing high levels of *Cr2* and low levels of *Ighd*, exclusively identified in the spleen, are known as marginal zone (MZ) B cells [29] (Figures 2C,D and S5C). Conversely, mature B cells with high expression of *Ighd* and low expression of *Cr2* were categorized into nine subtypes (Figure S5D), representing distinct differentiation states and tissue heterogeneity. Among these B cells, we discovered that MZ B cells in GF mice exhibit a higher proportion of specifically differentially expressed genes (DEGs) compared to non‐MZ mature B cells, with upregulation linked to lymphocyte activation, B cell differentiation, and adaptive immunity. Conversely, downregulated genes are involved in cellular homeostasis, immune system development, apoptotic signaling, and cell cycle progression (Figure 2E). In the spleen, mature B cells primarily originate from splenic follicles, known as follicular (FC) B cells (subtype 5, Figure 2D). Both scRNA‐seq and Stereo‐seq data analysis of the spleen revealed a marked decrease in the absolute numbers of B cells in the GF mice (Figure 2F,G), which was closely associated with the upregulation of the *Cr2* gene expression (Figure 2D). The FC versus MZ B lymphocyte cell fate decision is regulated by *Cr2* [30]. The enhanced maturation of FC B cells was accompanied by the absence of MZ B lymphocytes and the downregulation of *Cr2* expression in FC B cells [30]. Therefore, the high expression of *Cr2* in GF FC B cells indicated impaired development and maturation of FC B cells. Thus, we infer that the microbiota modulates the development of splenic B cells by regulating *Cr2* gene expression, thereby influencing the innate‐like immune response of splenic MZ B cells and the adaptive immune function of FC B cells [29, 30]. Furthermore, we noticed impaired differentiation and activation of B cells in the GF gut. For example, the proportion of an activated B cell subtypes (subtype 7, with high expression of *Hspa1a, Hspa1b, Atf3*, and *Cd83*) exhibits a substantial reduction in the GF gut (Figure 2C). Concurrently, there was an elevated proportion of an unactivated B cell subtype (subtype 8) in the GF gut (Figure 2C). These results imply that the microbiota influenced the development of B cells in multiple organs.

Additionally, integrative analysis of B cells identified three subtypes of plasma cells, with IgA‐positive plasma cells being the predominant subtype in SPF mice, primarily distributed in blood (77.61%), liver (55.66%), and gut (79.44% in ileum, 65.46% in colon, and 81.14% in cecum) (Figures 2H, S5B and S6). IgG‐positive plasma cells were predominantly located in the liver (24.58%) and spleen (17.87%), while IgM‐positive plasma cells were primarily found in the spleen (61.37%). After the depletion of microbes, the proportion of IgA significantly decreased, while the proportion of IgM remained largely unchanged. This is consistent with previous studies, indicating that the majority of IgA production is microbiota‐dependent [31, 32], whereas IgM production is relatively independent of the microbial community, and can differentiate in a T cell non‐dependent manner [33].

### The microbiota exhibits organ‐specific heterogeneity in the regulation of myeloid cell function and T cell development and maturation

**Figure 3:**
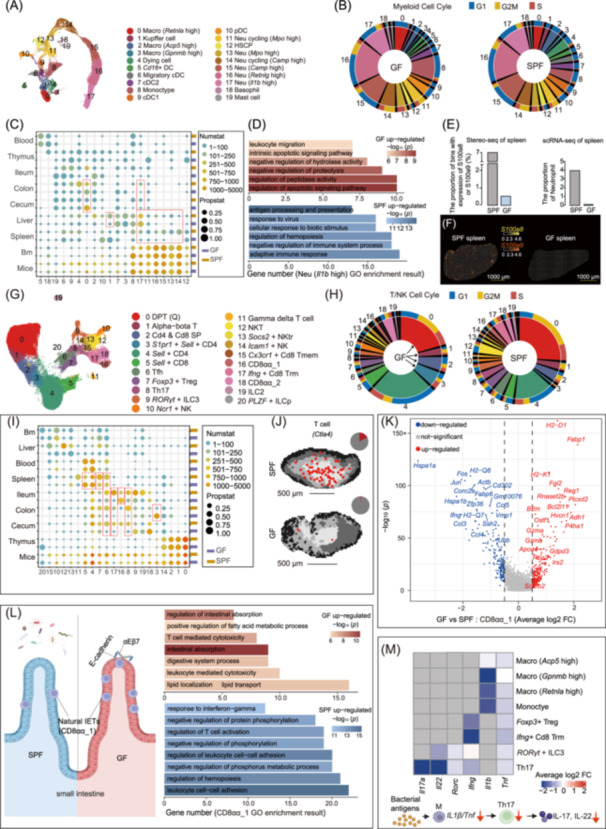
Microbiota‐regulated heterogeneity of the myeloid and NK/T cell compartment across tissues. (A) The UMAP visualizations of myeloid cell compartment. (B) The proportion and cell cycle of myeloid subtypes. (C) Heatmaps illustrating the subtype distribution of myeloid cells across each tissue and all tissues between GF and SPF mice. (D) Go enrichment analysis of neutrophils with high expression levels of *Il1b*. (E) The proportion distribution of neutrophils in the spleen of GF and SPF mice across scRNA‐seq and Stereo‐seq data. (F) The spatial visualizations of neutrophils marker genes in spleen. (G) The UMAP visualizations of NK/T cell compartment. (H) The proportion and cell cycle of NK/T subtypes. (I) Heatmaps illustrating the subtype distribution of NK/T cells across each tissue and all tissues between GF and SPF mice. (J) Spatial visualization of *Ctla4* gene of T cells in the white pulp region of spleen. (K–L) The volcano plot of CD8αα_1 differentially expressed genes (DEGs) (K) and functional enrichments of the top pathways (L) are shown. (M) Heatmap displaying the differential fold‐change (FC) of *Il17a*, *Il22*, *Rorc*, *Ifng, Il1b*, and *Tnf* across lymphoid subtypes. The color gradient in the heatmap represents the average FC values of GF versus SPF. FC > 0 indicates up‐regulated expression in GF mice. A simplified diagram illustrates the activation of Th17 cells by macrophages acting as antigen‐presenting cells (APCs).

For myeloid cell compartments, our cross‐tissue analysis identified 20 distinct subtypes (Figures 3A−C and S7A,B). We observed many DEGs in myeloid cells, with the most significant changes being the macrophages subtype observed in the large intestinal (subtype 0, *Retnla* high macrophage, Figure S7C). In SPF mice, these macrophages primarily focus on antigen presentation, regulation of T cell activation, and adaptive immune response. In GF mice, they are enriched in ERK1/2 signaling, endocytosis, wound healing, chemotaxis, and leukocyte migration (Figure S7D). The significantly increased proportion of these macrophages in the cecum of GF mice may be related to the maintenance of homeostasis in the enlarged cecum (Figure 3C). These findings unveil the significant impact of the microbiota on the functions of macrophages and their plasticity under varying environmental conditions. Furthermore, in GF mice, the upregulated genes in bone marrow neutrophils are associated with protein folding during development and apoptosis during maturation (Figures 3D and S8A). This suggests that the absence of the microbiota may result in reduced neutrophil survival. The decrease in neutrophils in peripheral organs (Figures 3E−F, S7B, and S8B), including the liver, spleen, and intestine, could be associated with developmental abnormalities within the bone marrow.

Twenty‐one distinct subtypes of NK/T cell compartments were identified (Figures 3G and S9A,B). In GF mice, the cell cycle of T cells at the early developmental state (subtype 0, 1, and 2) within the thymus was dysregulated (Figure 3H), with the highest number of DEGs predominantly enriched in pathways closely associated with T cell activation and differentiation (Figures S9C and S10A−D). These result indicate that the absence of microbiota can significantly impact the normal progression of T cell development and maturation within the thymus. Naive CD4^+^ T cells (subtype 3 and 4) and CD8^+^ T cells (subtype 5) with high expression of *Ccr7* and *Sell* increased markedly in GF mice (GF, 42.3% vs SPF, 23.4%) and were mainly found in gut, spleen, blood, and thymus (Figures 3I and S9A,B). Other mature CD4^+^ T cells including follicular helper T cells (Tfh) expressing *Cxcr5*, regulatory T cells (Tregs) expressing *Foxp3* and *Ctla4*, and tissue‐resident Th17 cells expressing *Il17a* and *Il22* were found largely decreased in the intestine (colon and ileum) and spleen (Figures 3I and S9A). The significant reduction in these specialized T cell subtypes in GF mice underscores the critical role of the gut microbiota in shaping the immune system maturation and in maintaining immune homeostasis (Figure 3I,J). Additionally, we found four intraepithelial T cells (IETs) including two unconventional double‐negative (DN) thymocytes subtypes (subtype 16 and 18), one conventional single‐positive (SP) thymocytes subtype (subtype 3), and one αEβ7^+^CD8^+^ recent thymic emigrants (RTEs) subtype (subtype 17) [34] (Figure 3G). The naive conventional SP IETs with high *S1pr1* expression (subtype 3) found in the colon in our study are consistent with previous research [34] (Figure S10E,F). The proportion of such naive conventional SP IETs significantly increased in the colon of GF mice likely due to lack of stimulation by microbiota‐derived antigens. In contrast, the RTEs migrated exclusively into the small intestine reflecting the high expression integrin αEβ7 and were induced to produce IFN‐gamma (*Ifng*), defined as *Ifng* ^*+*^ CD8^+^ resident memory T cells (Trm) (Figures 3I and S10E,F). Integrin αEβ7 is crucial for effector T cell homing to the small intestine [35]. The proportion of such induced IETs was significantly reduced in GF mice reflecting the lack of stimulation by microbiota‐derived antigens. The natural IETs expressing integrin αEβ7 and showing a *Cd3d* ^+^ *Cd8a* ^+^ *Cd8b* ^–^ *Tcrg–C4* ^+^ *Trbc2* ^+^ signature were defined as CD8αα_1 (subtype 16) and CD8αα_2 (subtype 18), respectively [36, 37] (Figure S9A). Here, CD8αα_1 exhibited an increased expression of *Gzma* and *Gzmb* [38] (Figure S9A), with classical cytotoxic T cell characteristics, and with a notable increased proportion in the ileum of GF mice (Figure 3I). Interestingly, functions related to lipid absorption and T‐cell‐mediated cytotoxicity of CD8αα_1 cells in the ileum of GF mice were also significantly enhanced (Figure 3K,L and Table S5). Lastly, we defined ILC2 and RORγt^+^ ILC3 populations via expression of markers including *Gata3*, *Il13*, *Il22*, and *Rorc* (Figures 3G and S9A). Conflicting reports on the requirement of commensal bacteria for the development of RORγt^+^ ILC3 have been presented [39, 40, 41, 42, 43]. Our study identified a reduction in ILC3 cells and a lack of expression of *Rorc* or *Il22* in the ileum of GF mice (Figure 3I,M), consistent with previous reports [41, 42, 43]. Upregulated genes in ILC3s in GF mice were enriched in GO terms including leukocyte‐mediated cytotoxicity, cell killing, and response to lectin (Table S5). We observed that GF mice exhibited impaired expression of *Il1b* and *Tnf* in macrophages in the large intestine, and *Il17a* and *Il22* in Th17 and RORγt^+^ ILC3 cells (Figure 3M). Previous studies have reported that symbiotic bacteria stimulate the production of *Il22* and *Il17a* in intestinal Th17 cells and RORγt^+^ ILC3 cells by promoting a steady‐state expression of *Il1b* and *Tnf* in intestinal macrophages [44].

In order to investigate the gene expression changes of key factors in all lymphoid subsets under conditions of microbial depletion, we downloaded six immunologically relevant lists of genes from ImmPort database [45] referring to antigen processing and presentation, antimicrobials genes, chemokines, cytokine and receptors, interferons TNF and TGF‐beta family members, interleukins, and receptors. We observed significant downregulation of different regulatory factors from various cell types in GF mice, such as the downregulation of the antimicrobial genes *Slpi* in B/Plasma cells, *IL‐17A* and *IL‐22* in Th17 cells, *Ifng* in T/NK cells, *Isg15, Ccl5, Cxcl10* and *Ifih1* in T/NK/ILCs/macrophages, and *Il1b* in monocytes/macrophages. Conversely, there was an upregulation of the antimicrobial gene *Fabp4* and *Fabp5* in liver macrophages, *Cd14* in monocytes/neutrophils, *Ccr1* and *Ccl24* in cecal macrophages, *S100a8, S100a9,* and *S100a6* in Kupffer cells of liver and neutrophils of bone marrow, and *Cd209a* and *Il6* in cDC2/migratory cDC in GF mice (Figure S11).

### The microbiota regulates zonation, function, and nutrient absorption of intestinal mucosa

**Figure 4:**
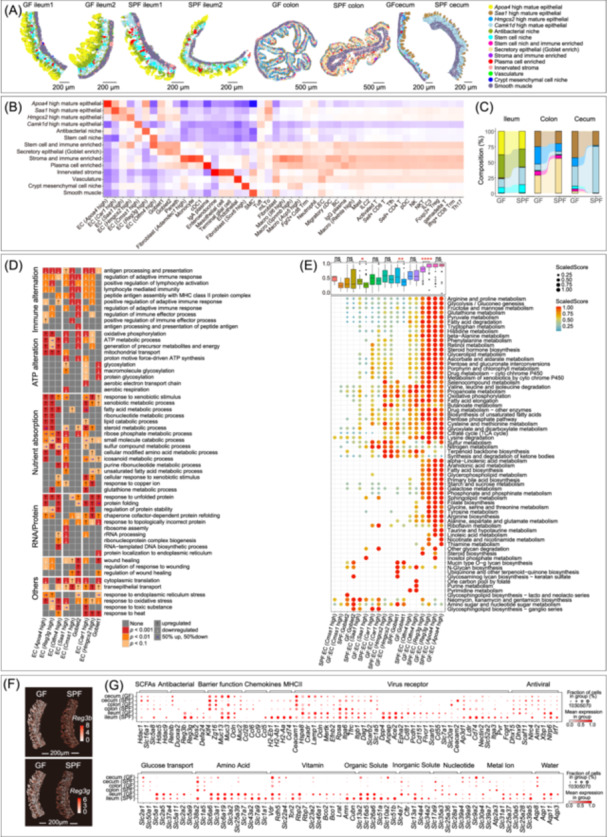
Microbiota depletion leads to alterations in the intestinal epithelial function and zonation. (A) Intestinal region zonations based on Stereo‐seq. (B) Heatmap visualizes log2‐transformed enrichment scores of cell types in tissue zones, with higher values indicating greater abundance. (C) The relative proportions of epithelial compartments. The first two zones of the histogram represent the region at the top of the intestinal epithelium. (D) Heatmap showing Gene Ontology enrichment analysis of DEGs in different intestinal segments. (E) Heatmap of metabolic pathway activity in different subtypes of intestinal epithelial cells. Activity scores were calculated with the scMetabolism package, normalized as (V_cur_ − V_min_)/(V_max_ − V_min_) per pathway and mouse, where V_cur_ is the current activity score, V_min_ is the lowest, and V_max_ is the highest. Statistical analyses of the scaled scores were performed using Wilcoxon tests. ns *p* > 0.05, *p < 0.05, **p < 0.01, ***p < 0.001, ****p < 0.0001. (F) Spatial visualization of the expression of innate defense genes *Reg3β* and *Reg3γ*. (G) Dot plot depicting the expression profiles of distinct functional genes across different segments of the intestine. Each dot signifies a gene, with color saturation denoting the average expression level (scaled by Z‐score) within a specific intestinal segment. The size of each dot corresponds to the proportion of cells expressing the respective gene.

We next explored microbial effects on intestinal segments, focusing on the epithelial layer, through spatial deconvolution of Cell2location using integrated intestine subtypes data (Figure S12). A total of 14 zonations were identified in the gut (Figure 4A,B). Both scRNA‐seq and Stereo‐seq data analysis showed significant changes in epithelial zonation in GF mice compared to SPF mice, including thicker apical absorption zone in the ileum (*Apoa4* high mature epithelium), colon (*Saa1* high and *Hmgcs2* high mature epithelium), and cecum (*Saa1* high and *Hmgcs2* high mature epithelium), but thinner secretory zone (Goblet cell enriched) in the colon of GF mice (Figure 4C). Consequently, reflecting the close contact with the microbiota, intestinal epithelial zonations are strictly regulated by the microbiota.

Differential gene enrichment analysis of gut epithelial subtypes revealed the immature adaptive immunity, particularly in the colon of GF mice (Figure 4D). Three epithelial compartments of the ileum in GF mice exhibited a substantial upregulation of expression of genes involved in energy metabolism‐related and nutrient absorption functions, while the colon and cecum epithelium exhibited downregulation of these functions (Figure 4D and Table S6). The increased expression of genes involved in nutrient absorption and energy metabolism and decreased expression of genes involved in immune responses in the ileum are consistent with previous studies [46]. This situation mirrors previously reported findings that intestinal epithelial cells can focus on metabolic functions if the immune system functions optimally [46].

More detailed analyses of metabolic pathways utilizing the scMetabolism software package revealed functional heterogeneity regarding metabolic activity among different subtypes of intestinal epithelial cells. Absorption of nutrients and accompanying metabolic activity in the gut of mice are mainly concentrated in the ileum, especially in the upper villous area of the intestinal mucosa. Meanwhile, our metabolic pathways analysis indicates that GF mice exhibit enhanced metabolic activity in the submucosal region where *Reg3g* is enriched (Figure 4E). Members of the C‐type lectin *Reg3* gene family encode peptides with antibacterial effect and are expressed in intestinal epithelial cells. However, in GF mice, the expression of *Reg3* (*Reg3b* and *Reg3g*) is severely reduced but shows an enriched expression pattern at the bottom of the mucosa demonstrating functional compartmentalization (Figure 4F).

We subsequently extended our analysis to 340 regulatory genes or receptor genes closely associated with bacteria, viruses, solute carrier (SLC) transporters, vitamin absorption, and water absorption in the intestinal epithelium (Table S7). We found that MHC II‐related genes were almost not expressed in intestinal epithelial cells in GF mice (Figure 4G). Interestingly, the expression of antiviral‐related genes (such as *Dhx15, Dhx9, Scaf11, Nlrp6, Hspa8, Lamp1, Mertk, Dag1, Scarb1, Cd81, Cd151, Pcdh1*, and *Slc7a1*) in the ileum were upregulated in GF mice (Figure 4G). As far as intestinal epithelial immunity is concerned, it appeared that GF mice have adaptive immune deficiencies associated with antigen processing and presentation, while innate immune activation associated with antiviral activity was maintained in GF mice. Furthermore, we observed upregulation of the *Vdr* gene in GF mice in all intestinal segments, which is consistent with previous research [47]. The upregulation may reflect an attempt to maintain or enhance the intestinal barrier function, especially in the absence of the microbiota [48].

### The microbiota regulates dietary lipid processing by intestinal enterocytes and regulates blood lipid levels by ZBTB20‐LPL axis in the liver

**Figure 5:**
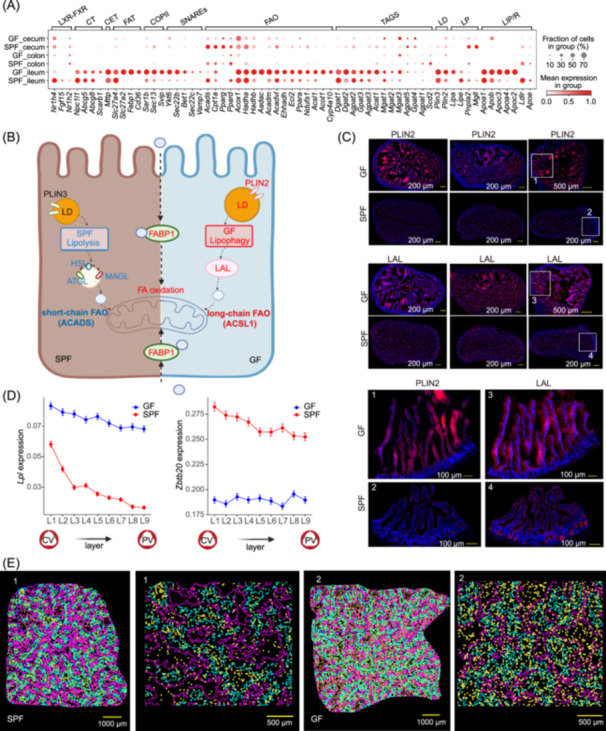
The altered landscape of lipid absorption and metabolism in intestinal epithelial cells and the liver spatial zonation feature of blood lipid regulation axis of ZBTB20‐LPL following microbial depletion. (A) Dot plot depicting the gene expression profiles linked to lipid absorption and metabolism. CET, chylomicron transport; CT, cholesterol Transport; FAT, fatty acid transport; COPII, coat protein complex II; SNAREs, the soluble N‐ethylmaleimide‐sensitive factor (NSF) attachment protein (SNAP) receptor; FAO, fatty acid oxidation; TGS, TAG synthesis; LD, lipid droplet; LP, lipolysis; LIP/R, lipoprotein and receptor. (B) A simplified diagram illustrates the LD storage, lipolysis, and lipophagy processes between SPF and GF mice. (C) Immunofluorescence staining of ileum. (D) The proportions of bins expressing the *Lpl* and *Zbtb20* genes are shown for GF and SPF mice in each layer. CV, central vein; PV, portal vein. (E) Spatial visualization of *Lpl* gene expression in the liver. The background represents the layers of the liver. [Correction added on 17 February 2025, after first online publication: Figure 5 was updated with the revised one.]

The altered intestinal epithelial zonation in GF mice, particularly the thickening of the apical nutrient absorption zone, underscores the impact of the intestinal epithelium on diet‐mediated lipid absorption and metabolism. Meanwhile, we observed that DEGs of intestinal epithelial cells were also related to basic physiological characteristics in human, such as blood lipids and BMI (Table S8). Then, we assessed mRNA expression of 69 genes across different gut segments involved in fat absorption, pre‐chylomicron processing in the endoplasmic reticulum (ER), and maturation in the Golgi apparatus for entry into circulation (Table S9). In GF mice, we observed significant upregulation of *Cd36* mRNA in ileum epithelial cells, indicating a potential for increased fatty acid uptake, consistent with reduced uptake in *Cd36*‐deficient mice [49] (Figures 5A and S13). This upregulation coincided with enhanced expression of triacylglycerol (TAG) synthesis enzyme‐encoding genes (*Gpat3, Mgat2, Dgat2*, and *Acat1*), suggesting microbiota‐dependent regulation of ER‐associated lipid synthesis. High mRNA levels of *Apob* and *Apoa4* were also detected during chylomicron packaging, with elevated *Sar1b* (a key part of the COPII coat complex), *Fabp1*, *Bet1*, and *Ykt6* mRNA implicating altered intracellular transport mechanisms from the endoplasmic reticulum to the Golgi apparatus (Figure S13). In addition, chylomicrons can be stored via lipid droplet (LD) formation, and the LD surface proteins PLIN2 and PLIN3 interact with LDs, regulating their formation, stability, and metabolism, influencing intracellular lipid storage and utilization. PLIN2 is more common on the largest LDs [50]. Consequently, the elevated expression of PLIN2 in GF mice implies an augmentation of large cytoplasmic LDs in the absence of the gut microbiota. Concurrently, the decreased expression of lipolysis genes (ATGL, *Pnpla2*; HSL, *Lipe*; MAGL, *Mgll*) coupled with an increase in LAL (*Lipa*) mRNA indicates that lipophagy, an alternative lipid processing mechanism, is the primary mode of LDs degradation in GF mice (Figure 5B). Protein fluorescence staining further confirms the distinct LD storage and lipophagy processes in GF mice (Figure 5C). Furthermore, compared to SPF mice, GF mice exhibited upregulation of genes involved in medium‐ and long‐chain fatty acid oxidation, such as *Ppara, Fabp1, Acadl, Acadm, Acox1, Hadha*, and *Hadhb*. Concurrently, there is a downregulation of genes involved in the oxidation of short‐chain fatty acids, including *Acads* (Figure 5B). This indicates a shift in lipid metabolic pathways in the absence of microbiota, which could be an adaptive response to the altered lipid environment in GF mice. Taken together, the absence of microbiota triggers coordinated dynamics in lipid absorption, transport, chylomicron synthesis, LD formation, lipolysis, and fatty acid oxidation within the enterocytes of the small intestine.

In microbial depletion, lipid metabolism in the small intestine is disrupted. Whether this impacts liver metabolism or is modulated by pivotal hepatic factors is still unclear. To investigate this, we first analyzed differential gene expression across liver zones: peri‐portal, mid‐portal, and central vein (Details were shown in Methods). Key findings include enrichment in lipid synthesis, transport, homeostasis, chylomicron remnant clearance, and triacylglycerol metabolic regulation pathways (Figure S14A,B). Then, we measured RNA levels of genes encoding 75 enzymes involved in FA synthesis and oxidation, TAG synthesis, and lipolysis in hepatocytes (Table S9). We observed that the expression level of these genes were generally downregulated in the GF liver (Figure S14C). A schematic diagram of hepatocytes summarizing these processes is presented in Figure S14D. Briefly, expression of genes involved in fatty acid, TAG synthesis (*Scd1, Elovl3, Dgat2, Agpat2, Agpat3, Gpat4*, and *Plin2*), and FA oxidation (*Ppara, Acadl, Acadm, Acox1, Cpt1a, Acads, Eci2, Aadac, Acot1, Cyp4a10, Hadha, Hadhb*, and *Ehhadh*) in the liver of GF mice was significantly down‐regulated. According to previous reports, FXR promotes mitochondrial β‐oxidation by activating PPARα, enhances cholesterol uptake through induction of LDLR and syndecan‐1, and triggers *Fgf15* synthesis in the intestine to activate the ERK1/2 pathway in hepatocytes, leading to the inhibition of *Cyp7a1* and BA synthesis [51]. The expression levels of these genes in our study are consistent with the previous reports [51] (Figure S14D). However, confirming this regulatory relationship requires additional experimental validation.

Furthermore, we observed significant downregulation of the zinc finger transcription factor (*Zbtb20*) in the livers of GF mice, accompanied by an increase in lipoprotein lipase (*Lpl*), reflecting that *Zbtb20* suppresses *Lpl* gene transcription by directly binding to its promoter [52] (Figure S14E). LPL, a key enzyme in lipid metabolism, hydrolyzes circulating triglycerides. It is quickly transported by GPIHBP1 to the endothelial cell surface, facilitating TAG hydrolysis [53] (Figure S14E). Due to the minimal expression of *Lpl* in adult livers, it has often been overlooked. Our study reveals that in SPF mice, *Lpl* expression is predominantly localized to the central vein regions, and its expression markedly increases following microbial depletion, without exhibiting hepatic zonation distribution characteristics (Figure 5D,E). Interestingly, specific deletion of hepatic *Lpl* resulted in a significant decrease in plasma LPL content and activity, impaired postprandial TAG clearance, and increased plasma TAG and cholesterol levels, but without affecting liver lipids or glucose homeostasis [54]. In addition, we note that the decreased *Ldlr* expression and increased *Lpl* expression may be contributing factors to the elevated levels of low‐density lipoprotein in the blood of GF mice (Figures 1B and S14E).

The simultaneous alterations in liver lipogenesis and lipid oxidation gene expression, along with enhanced FA oxidation and ileum lipophagy in GF mice, likely contribute to their resistance to high‐fat diet‐induced obesity.

### Integrative analysis reveals that depletion of the microbiota alters the synthesis pathway and reabsorption capacity of BAs

**Figure 6:**
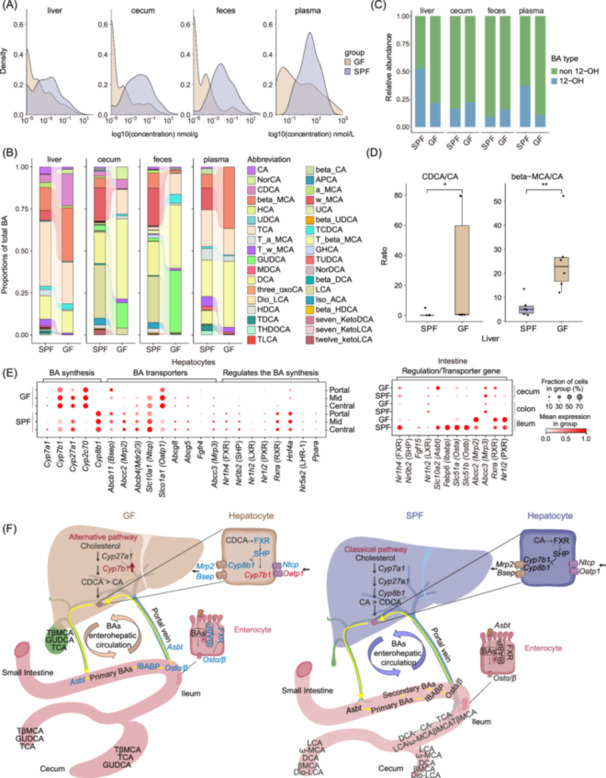
Microbiota depletion alters the synthesis pathway and reabsorption capacity of BAs. (A) We plot the density distribution of concentration values to illustrate the differences in concentration kurtosis between GF and SPF mice in the various body sites. For solid samples, the concentration values are expressed in nmol/g, and for liquid samples, the concentration values are expressed in nmol/L. (B) Bar plot shows the proportions of BAs in different samples, including liver, cecum, feces, and plasma between GF and SPF mice. Each colored area represents a type of BA and is stacked along the x‐axis to display their changes between the two mouse groups. Alterations are depicted through variations in height and thickness of the different color areas and are highlighted by the streamline. APCA, apocholic acid; CA, cholic acid; CDCA, chenodeoxycholic acid; DCA, deoxycholic acid; Dio‐LCA, 7,12‐Diketolithocholic acid; G, glyco; HCA, hyocholic acid; HDCA, hyodeoxycholic acid; Iso‐LCA, Isoallolithocholic acid; LCA, lithocholic acid; MCA, muricholic acid; NorCA, norcholic acid; NorDCA, nordeoxycholic acid; T, tauro; UCA, ursocholic acid; UDCA, ursodeoxycholic acid; 3‐oxoCA, 3‐Dehydrocholic acid; 7‐KetoLCA,7‐ketolithocholic acid. (C) Bar plot showing absolute contents of non‐12 OH and 12‐OH BAs in different samples. The category of 12‐OH BAs encompasses CA, DCA, and other BAs originating from the classic pathway, while non‐12 OH BAs include CDCA, MCA, and other BAs derived from the alterative pathway. (D) Boxplots showing the ratios of CDCA to CA and β‐MCA to CA in the liver. The points represent each sample, while the horizontal lines depict the median and the boxes indicate the 25th−75th percentile values. Differences between groups were analyzed by the Wilcoxon test. *p < 0.05, **p < 0.001. (E) Dot plot depicting the gene expression profiles linked to BA metabolism in the liver and BA absorption in the intestines. (F) A cartoon illustrates the hepato‐intestinal axis, showing molecular processes of BA circulation, with genes in blue indicating downregulation and genes in red indicating upregulation in GF mice.

BA metabolism, modulated by the gut microbiota, plays a key role in regulating glucose and lipid metabolism and maintaining energy balance. To investigate the influence of microbial depletion on BAs composition, we specifically focused on the overall levels of BAs (Figure 6A), and provided detailed information of each BA in different niches (Figure S15A). Overall, GF mice exhibited reduced levels of BAs in multiple organs, including liver and cecum, and in feces and plasma, consistent with previous findings [17]. In GF mice, the liver predominantly contained free primary BAs, including chenodeoxycholic acid (CDCA) synthesized through the alternative pathway, and β‐muricholic acid (β‐MCA), which constituted approximately 50.9% of total BAs. Conversely, SPF mice exhibited a liver composition comprising mainly conjugated primary BAs, such as taurocholic acid (TCA) synthesized via the classical pathway, and tauro‐β‐muricholic acid (T‐β‐MCA), accounting for about 52% of total BAs (Figure 6B). Notably, GF mice showed a significant increase in the proportion of non‐12‐OH BAs (Figure 6C), particularly CDCA and β‐MCA, along with concurrent elevation in the CDCA/CA and β‐MCA/CA ratios in the liver (Figures 6D and S15B). These findings suggested that microbial depletion not only impacts the composition of BAs in terms of their free and conjugated forms but also induces alterations in the synthesis pathway of BAs, transitioning from the classical synthetic pathway to alternative pathways in the liver.

To investigate changes in gene expression related to BA synthesis, we conducted a survey on the expression patterns of five key genes (*Cyp27a1*, *Cyp7b1*, *Cyp7a1*, *Cyp8b1*, and *Cyp2c70*) using Stereo‐seq data of hepatic zonation (Table S10). We observed a significant increase in the expression of *Cyp7b1* and *Cyp2c70* throughout all liver layers in GF mice (Figures 6E and S15C). Conversely, the expression of *Cyp8b1*, an important enzyme in the classical pathway, was reduced, consistent with previous research [55, 56] (Figure 6E). These results indicated a shift in the balance of key enzymes between the classical and alternative pathways, further confirming the utilization of alternative pathways for BA synthesis in the liver of GF mice (Figure 6F).

The absence of microbiota affects more than BA production, it also crucially regulates BA circulation through interactions with reabsorption transporters. In GF mice, we observed a significant reduction in the expression of two key genes, *Bsep* and *Mrp2*, which are responsible for transporting conjugated BAs and facilitating their storage in the gallbladder (Figure 6E). Most genes encoding BA transporters exhibited diminished expression in the livers of GF mice, especially in the vicinity of the hepatic portal vein, which is the liver region most affected by the lack of microbiota (Figure 6E). Moreover, in the intestinal tract of GF mice, we observed changes in the expression of genes encoding BA transporters. For instance, the expression of *Asbt*, a membrane protein expressed on the microvilli of enterocytes in the small intestine and responsible for re‐uptaking deconjugated BAs from the intestinal lumen into the enterocytes, was reduced in the ileum and increased in the cecum of GF mice. Furthermore, *OSTα/β*, encoding key transporters involved in transporting BAs from the gut back to the liver via the hepatic portal vein, also showed reduced expression in the ileum, where deconjugated BA reuptake occurs. A schematic diagram summarizing molecular processes of BA synthesis and hepato‐intestinal axis circulation in SPF and GF mice is presented in Figure 6F. Interestingly, the livers of GF mice exhibited an increased proportion of cholangiocytes as compared with the SPF counterpart, demonstrated by Stereo‐seq, scRNA‐seq data, and HE staining (Figure S16A–F). This result is consistent with previous findings of increased vascularized area in GF mice [57]. Gene Ontology (GO) analysis of differential genes in cholangiocytes revealed that upregulated genes in GF mice are associated with pathways related to oxidative stress, wound healing, and apoptotic signaling (Figure S16G). This phenomenon may be attributed to metabolic and physiological changes resulting from the lack of gut microbiota, thereby affecting the structure and function of the bile system.

## DISCUSSION

Identical feeds were administered to both GF and SPF mice, starting from the third week post‐weaning and continuing into adulthood (Table S1). Therefore, the comparative omics data between GF and SPF mice reflect the impact of microbial absence on the molecular level of host organs. Our analytical approach encompassed changes from macroscopic tissue structure to fine structure, alterations from fine structure to cellular composition, transitions from cellular levels to molecular distinctions, and tissue heterogeneity related to cellular differences. Thus, our data set provides a valuable omics resource for the study of the development and regulation of the immune system and metabolic regulation.

In terms of immune homeostasis, our cross‐organ analysis revealed several important features. First, microbial deficiency resulted in abnormal development of B, myeloid, and T/NK cells, encompassing (1) a perturbed cell cycle progression and activation and differentiation function in developing immune cells, (2) an increased proportion of naive immune cells and decreased proportion of mature immune cells in GF mice, (3) a decreased B‐cell count, immature follicular B cells, as well as the abnormal differentiation and proliferative functions of MZ B cells in the spleen of GF mice, (4) the abnormal apoptosis of neutrophils in the bone marrow and a substantial decrease in peripheral tissues in GF mice, (5) the shift in macrophage role from antigen presentation and T cell regulation in SPF mice to *ERK1/2* signaling and wound healing in GF mice, suggesting the microbiota's role in macrophage function and plasticity, (6) an immature development of intraepithelial‐induced T cells in the colon, and (7) an increased proportion of intraepithelial natural T cells and enhanced lipid absorption potential in the ileum of GF mice. As the central immune organ, the thymus exhibited similar cellularity in both SPF and GF mice in our study. But of note, we revealed a decrease in the proportion of developmental T cells in the G2M phase of GF mice. This indicates that microbial deficiency may prolong the cell cycle time and reduce proliferation of developmental T cells in the thymus resulting in a steady‐state that is different from that of conventional animals. This result is consistent with previous conclusions on the proliferative activity in colonic crypts of GF mice [58]. Nevertheless, further investigations are needed to elucidate the specific mechanisms through which microbes regulate the cell cycle of the developing T cells in the thymus and their role in maintaining immune homeostasis. Second, the immune cell aggregation index demonstrated cell and tissue heterogeneity. Compared to other immune cells, plasma cells have the highest degree of aggregation, and the aggregation area in the intestine is larger than in other tissues. The aggregation area of plasma cells in the gut of GF mouse was smaller than that in SPF mice. The difference in the level of IgA in the mouse intestine may be related to the strength of antigens, as GF mice primarily encounter dietary antigens. Furthermore, IgA restricts bacterial access to the epithelium and to intestinal immune cells [46, 59, 60], and it can change gene expression in bacteria [61] or promote survival of specific bacteria [46, 62]. The absence of microbiota led to a significant decrease in the proportion of plasma cells in most tissues, except thymus [21, 24, 25, 26]. Third, a decreased adaptive immunity capacity including cell proportion and lack of expression of crucial molecules in GF mice was found, including a decreased percentage of regulatory T cells in cecum, colon and spleen, follicular helper T cells in cecum, colon, ileum and spleen, toxic *Ifng* ^*+*^ CD8^+^ T in ileum, Th17 cell in the large intestine, and the dramatically decreased expression of key immune cell genes, such as *Il17a, Il22*, and *Ifng*.

BA metabolism is a complex process involving the conversion of cholesterol in the liver, facilitating the consumption of cholesterol, and the absorption of fat in the food. In this study, we integrated multi‐omics data to elucidate the impact of microbial depletion on the synthesis, metabolism, and circulation of BAs, processes with significant implications for human health. Our findings demonstrated as expected a reduction in secondary BA levels in GF mice, accompanied by a shift from the classical pathway to the alternative pathway. This shift is attributed to changes in the expression of key genes involved in BA synthesis in GF mice. Specifically, we observed an increase in the expression of *Cyp7b1*, which controls the alternative pathway, and a decrease in the expression of *Cyp8b1*, which is important for the classical pathway. Microbial depletion not only affects BA synthesis, but also exerts a significant impact on the regulation of BA transporters and the enterohepatic circulation. In GF mice, we observed reduced expression of various genes encoding BA transporters, including *Bsep* and *Ntcp* in the liver, as well as alterations in expression of genes encoding transporter in the intestinal tract. These findings suggest that the microbiota plays a crucial role in modulating BA absorption, recycling, and distribution within the body. The spatial distribution variations in gene expression within the liver and intestinal tract further highlight the intricate relationship between the microbiota and BA metabolism. These findings provide valuable insights into the influence of gut microbiota on BA metabolism and gene expression regulation. Moreover, the biliary duct area in GF mice becomes larger, which is consistent with previous findings of an increased vascular area in GF mice [57] (Figure S16F). However, the relation between bile duct enlargement and perturbation of BA homeostasis remains to be further explored.

Stereo‐seq analysis of gut revealed that symbiotic bacteria impact on the functional intestinal epithelial zonation, with an increased functional area in the nutrient absorption zone of the ileum. Consistent with this, single‐cell integration analysis of the intestinal epithelium in different gut segments revealed significant enhancements in the ileum of GF mice in terms of expression of genes involved in fatty acid absorption, TAG synthesis, CE assembly and Golgi transport, LD formation, lipolysis, and lipid oxidation. Intriguingly, expression of genes involved in lipogenesis and oxidation processes in the liver exhibited an opposite trend of those in the ileum, with gene expression being reduced in the liver. The concomitant changes observed in expression of genes involved in lipogenesis and lipid oxidation in the liver and ileum of GF mice may represent a mechanism by which the organism maintains lipid metabolic balance. The lipophagy process in the ileal epithelium of GF mice can be considered a cellular self‐regulatory mechanism, playing a role in the adaptation to diverse physiological and environmental conditions. The microbial regulation not only affected the zonation of immune cells but also influenced the zonation of gene expression related to lipid metabolism in the liver (Figure 5D,E). Nevertheless, the physiological significance of this regulation of lipid metabolism requires further experimental validation.

It is very regrettable that, at present, spatial transcriptomics is still unable to achieve a high gene capture rate, which limits the spatial localization of immune cell subtypes and analysis of the surrounding microenvironment. In the future, with the advancement of spatial technologies, a plethora of algorithms tailored for spatial transcriptomics and in‐depth bioinformatics mining will continue to enhance our contributions to this field of research.

Even though we did not sample the entire gastrointestinal tract in both GF and SPF mice, and lack multi‐omics information on lymph nodes, lungs, and skin, we still envisage that our study provides a valuable background for investigating the intricate reciprocal crosstalk between host and microbiota. It should also be noted that our study did not explore the impact of different diets on immune homeostasis, lipid metabolism homeostasis, and BA homeostasis in GF and SPF mice. Further research on these aspects is clearly warranted. We envisage that this integrated multi‐omics and multi‐organs data set based on GF and SPF mice constitutes a useful resource facilitating the study of the mechanisms underlying immune and metabolic regulation and will enhance our understanding of the crosstalk between microbes and hosts.

## CONCLUSION

Our study presents a multi‐organ single‐cell, spatial transcriptomics, and BA omics atlas of SPF and GF mice. Plasma cell aggregation displays significant tissue heterogeneity depending on the gut microbiota. GF mice exhibit impaired follicular and marginal zone B cell maturation, linked to microbiota‐mediated modulation of *Cr2* gene expression. The microbiota regulates the development and survival of neutrophils in the bone marrow, influences the development and differentiation of T cells in the thymus, and modulates intraepithelial γδ T cell composition and lipid absorption in the small intestine. Notably, spatial transcriptomics of the gut have, for the first time, revealed significant changes in the functional zonation of the intestinal mucosa in GF mice, with an enlargement of LDs in absorptive enterocytes of the small intestine, transitioning toward lipophagy and enhanced fatty acid oxidation, underscoring a key pathway in GF mice's resistance to obesity. Concurrently, spatial transcriptomics of liver have also, for the first time, revealed that the zinc finger and BTB domain‐containing protein (ZBTB20)‐ Lipoprotein lipase (LPL) (ZBTB20‐LPL) axis, which regulates blood lipids, exhibits a gradient expression pattern in the liver that is modulated by microbes.

## METHODS

### Mice and tissues collection

Thirty‐six adult male SPF and GF Kunming (KM) mice were obtained from the Experimental Animal Center of Huazhong Agricultural University, Wuhan, China. They were fed the same diet for 7 weeks after weaning at 3 weeks of age. The feed had low crude fiber content (1%−4%) and was completely sterilized by 50 kGy of Co60‐g irradiation [63]. Table S1 lists the nutritional composition of the diets.

### Feces collection

Mice were fasted for over 12 h and feces were collected from sterilized cages the next morning. Cages were sterilized using high‐temperature and high‐pressure at 121°C and 100 kPa, followed by ultraviolet (UV). Fecal samples were transferred into sterile tubes, quickly frozen in liquid nitrogen for 15 min, and stored at −80°C. We collected fecal samples from 6 GF and 6 SPF mice for BA analysis.

### Serum isolation

Mice were anesthetized with pentobarbital sodium (60 mg/kg) via intraperitoneal injection before weighing and subsequent blood collection from the retro‐orbital venous plexus. Blood was left at room temperature for 1−2 h, then centrifuged at 3000 rpm for 15 min to separate serum, which was aliquoted and stored at −80°C. We successfully collected serum from 6 GF and 6 SPF mice for BA identification and conducted physiological and biochemical studies on 11 GF and 10 SPF mice. Additionally, whole blood for scRNA‐seq was collected from 2 GF and 2 SPF mice (Tables S2, S3).

### Bone marrow cell separation

To collect bone marrow, 10 mL DMEM was used to flush the marrow from the thigh bone and tibia into a sterile syringe. The cells were filtered through a 40 μm strainer into a 15 mL centrifuge tube, then centrifuged at 3000 rpm for 5 min at 4°C. The supernatant was aspirated, and the pellet was resuspended in 1 mL phosphate‐buffered saline (PBS) and 3 mL red blood cell (RBC) lysis buffer, incubated for 5 min at room temperature. After adding 8 mL PBS, the cells were centrifuged again at 3000 rpm for 5 min at 4°C, and this wash step was repeated twice. The final bone marrow cell suspension was diluted in PBS with 0.04% BSA. Bone marrow from 2 GF and 2 SPF mice was successfully collected for scRNA‐seq (Table S3).

### Tissue collection

Mice were euthanized via cervical dislocation, and sterile instruments were used for dissection. Tissues collected in order: bone marrow, spleen, ileum, colon, cecum, liver, and thymus. Except bone marrow, tissues were rinsed, dried, weighed, minced into 0.5 mm^3^ pieces, and stored in MACS Tissue Storage Solution for single‐cell analysis. At least 2 GF and 2 SPF mice per tissue were used for scRNA‐seq. For Stereo‐seq analyses, adjacent or corresponding tissues were embedded optimal cutting temperature (OCT) compound (Table S3). Briefly, tissues were snap‐frozen in precooled TissueTek OCT on dry ice, then stored at −80°C. A liver lobe was cut, frozen in liquid nitrogen, and stored at −80°C for BA analysis, with 6 GF and 6 SPF liver samples collected for targeted BA identification (Table S3). Procedures were completed within 20 min to ensure omics experiment success.

### Cecal content collection

All mice were euthanized as described above, and we squeezed the cecal contents into a sterile cryopreserved tube. These tubes were also rapidly frozen in liquid nitrogen for a duration of 15 min and then stored at −80°C. 6 GF and 6 SPF mice were successfully included for targeted BA identification (Table S3).

### Cell isolation for scRNA‐seq

Within 24 h, fresh blood and bone marrow samples were dissociated, and within 36 h, other tissues (spleen, thymus, cecum, colon, ileum, and liver) were processed at BGI‐QingDao's Single Cell Laboratory in Qingdao, China. Successful dissociation criteria included over 100,000 live cells, less than 10% clumping, and viability above 80%. To achieve this, cells were obtained from fresh tissue by enzymatic digestion. In brief, suitable small tissue blocks (no necrotic foci/no hemorrhagic foci/less fibrous connective tissue) were enzymatically digested with an enzymatic mixture containing 0.05% trypsin (Invitrogen, USA), 0.4% collagenase IV (Invitrogen, USA), 0.25% collagenase I (Sigma, USA), 0.13% collagenase II (BBI, China), and 0.1% elastinase (Worthington, USA) at 37°C for 30–45 min with agitation. After this, tissue digestion was terminated by adding 3 mL of FBS. The dissociated cells were subsequently passed through a 70 or 40 µm cell‐strainer (BD, USA) and centrifuged at 300 × *g* for 10 min. After the supernatant was removed, the pelleted cells were suspended in red blood cell lysis buffer (Thermo Fisher, USA) and incubated on ice for 3 min to lyse red blood cells. After washing twice with PBS (Invitrogen, USA), the cell pellets were re‐suspended in PBS (containing 0.04% BSA).

### Single‐cell RNA library construction and sequencing

The DNBelab C Series system was used for generating scRNA‐seq libraries, involving droplet encapsulation, bead collection, reverse transcription, cDNA amplification, and purification. cDNA was sheared into 250−400 bp fragments, and indexed libraries were made following the protocol. Quantification used the Qubit ssDNA Assay Kit and Agilent Bioanalyzer. Libraries were sequenced on the MGI DNBSEQ‐Tx with paired‐end sequencing, including a 30 bp read for barcodes and UMI, a 100 bp read for cDNA, and a 10 bp barcodes read for sample index.

### Single‐cell RNA raw data processing (alignment and UMI counting)

The sequencing data were processed using an open‐source pipeline (https://github.com/MGI-tech-bioinformatics/DNBelab_C_Series_HT_scRNA-analysis-software/tree/version2.0). Briefly, all samples underwent sample de‐multiplexing, barcode processing, and single‐cell 3' unique molecular identifier (UMI) counting with default parameters. Processed reads were then aligned to the reference genome (mm10) using STAR (v2.5.3) [64]. Valid cells were automatically identified based on the UMI number distribution of each cell by using the “barcodeRanks()” function of the DropletUtils tool to remove background beads and the beads that had UMI counts less than the threshold value. Finally, we used PISA to calculate the gene expression of cells and create a gene x cell matrix for each library.

### Spatial transcriptome experiment, library construction, and sequencing

The Stereo‐seq platform was utilized for spatial transcriptomics, with an established pipeline [65]. RNA quality was assessed with an RNA integrity number (RIN) threshold > 7. Tissue permeabilization was tested with varying durations (3, 6, 12, 18, and 24 min) on the Stereo‐seq Chip P Slide using 10 µm tissue sections, with a positive control of mouse brain tissue. The optimal permeabilization time, balancing strong fluorescence signal and minimal diffusion, was determined to be 12 min for spleen and liver, 18 min for thymus, and 6 min for ileum, colon, and cecum.

Post cryosection, tissue sections were attached to Stereo‐seq chips, incubated at 37°C for 5 min, and fixed with 20°C methanol for 40 min. The chips were then stained with a nucleic acid dye for ssDNA visualization as per the manufacturer's protocol, and ssDNA images were captured using a Ti‐7 Nikon Eclipse microscope. For permeabilization, the tissue sections placed on the chip were permeabilized using 0.1% pepsin (Sigma, USA) in 0.01 mol/L HCl buffer at 37°C for 12/18/12/6/6/6 min for spleen/thymus/liver/ileum/colon/cecum, and then washed with 100 mL 0.13 saline‐sodium citrate (SSC) buffer supplemented with 0.05 U/mL RNAase Inhibitor. mRNAs captured by DNBs on the chip were reverse transcribed at 42°C overnight. The chips were washed twice with 0.1 × SSC after in situ reverse transcription and incubated in tissue removal buffer at 37°C for 30 min. The chips were finally washed once with 0.1 × SSC after the tissue removal buffer was removed, then the chips were immersed in the cDNA Release Mix overnight at 55°C to release the cDNA. The released cDNAs on the chip were further fragmented, amplified, and purified to generate a DNA nanoball (DNBs) library according to the manufacturer's protocol [65]. The final DNBs were loaded into the patterned Nano arrays and sequenced on the MGI DNBSEQ‐Tx sequencer (MGI, China).

### Stereo‐seq raw data processing (alignment and UMI counting)

The Stereo‐seq FASTQ data contains unique identifiers (25 bp CID and 10 bp MID) in the first read and cDNA sequences in the second read. We used the SAW workflow (https://github.com/BGIResearch/SAW) to process this data, which involves several key steps, including mRNA spatial location restoration, filtering, mRNA genome alignment, gene region annotation, MID (Molecule Identity) correction, expression matrix generation, and tissue region extraction. Two core tools in SAW accomplish these steps. We utilized the SAW software's mapping tool to align read1's CIDs with the Stereo‐seq chip's coordinates, allowing a single base mismatch. This resulted in “Valid CID Reads”, which were then supplemented with coordinate data. The subsequent “Clean Reads” were obtained by filtering out MIDs with polyA sequences and low‐quality bases (N or more than two bases with a score below 10), as well as mRNA with polyA. These clean reads were then aligned to the mm10 reference genome using protocol [64] to create BAM files. The BAM files were processed for gene annotation, de‐duplication, and gene expression analysis with SAW's count tool, where uniquely mapped reads were tallied and gene expression levels were adjusted based on MID.

The gene expression profile matrix was segmented into non‐overlapping N × N DNBs, with transcripts of identical genes consolidated within each BinN unit. The Stereo‐seq data was categorized into two bin sizes: bin 20 (20 × 20 DNB, ~10 μm) and bin 50 (50 × 50 DNB, ~25 μm). This led to the creation of a spatial coordinates‐gene expression matrix for further analysis, with bin sizes adjusted to suit the analysis requirements.

Finally, the generated bin50 data sets contained 7446 for GF spleen and 11,957 for SPF spleen, 17,351 for GF thymus and 44,450 for SPF thymus, and bin20 data sets of 511,205 for GF liver and 410,282 for SPF liver, 7693 for GF ileum and 8358 for SPF ileum, 23,254 for GF colon and 33,515 for SPF colon, 4113 for GF cecum, and 5519 for SPF cecum (Table S3).

### Cell‐type deconvolution and identifying discrete tissue zones by clustering

Cell‐type deconvolution across the gastrointestinal tract was performed by integrating Stereo‐seq data with scRNA‐seq gene expression using Cell2Location (v0.1.3) [66]. The deconvolution process was carried out at two distinct scales, bin20 and bin50, utilizing integrated single‐cell RNA sequencing data from the gut and annotation results covering all subclasses within the categories of intestinal epithelium, B cells, NK/T cells, myeloid cells, and fibroblasts.

Initially, we filtered out bad bins with counts of expressed genes less than 50 and 150 at two bin scales of bin20 and bin50, respectively. Subsequently, taking the integrated gut scRNA‐seq data as input, we fitted a Cell2Location model with hyperparameters N_cells_per_location=4 and detection_alpha=20 to obtain spatial cell abundance estimations. Finally, we identified tissue regions by clustering Stereo‐seq bin20 or bin50 data using the estimated cell abundance of each cell type. This involved constructing a K‐nearest neighbor (KNN) graph and applying Leiden clustering. The result of region clustering with a number of KNN neighbors set to 15 best matched the anatomical structure of the gut.

### Cell type abundance estimates for tissue zones

Here, we use the Cell2Location tissue zone clustering results to make cell type abundance estimates for each tissue zone in order to better understand the tissue organization and predict cellular interactions. The tissue zones enrichment scores were defined across ileum, colon, and cecum. This enrichment score represents the average cell type percentage within the average of the corresponding tissue zone cluster divided by the average cell type percentage for all cells in the whole gut tissue. The value of enrichment scores after log2 transformation was displayed in the heat map. A higher value of enrichment score indicated a higher cell type abundance. It is worth noting that spatial clustering based on deconvoluted expression matrices has a significant advantage in eliminating batch effects from different slides.

### Cell clustering and cell type identification

We converted count data into Seurat objects using the R package Seurat (v4.3.0). Low quantity cells and low‐expression genes were identified and filtered, including (1) cell with high mitochondrial percentage (>50% for gut or >15% for other tissues), (2) cell with the abnormal number of gene or UMI (gene feature <200 and UMI > 50,000), (3) low gene/feature counts across cell (<3), (4) removing pseudo‐doublets by DoubletFinder [67]. After filtering, we normalized sample data and identified 2000 variable genes using Seurat's vst method. Batch adjustment was applied to each tissue using the “FindIntegrationAnchors” function to create an integrated data set. We ensured no batch effect post‐adjustment for both GF and SPF mice. For integrated data, we performed graph‐based clustering with 30 PCs and visualized it via UMAP. Cell clusters were annotated using known and identified markers from “FindAllMarkers”. We further analyzed cell subpopulations within main clusters. Cell cycle phase scores were calculated for each cell using the CellCycleScoring package and categorized into S, G2/M, or G1/G0 phases.

### Spatial aggregation index of immune cell

Based on the immune cell gene set scores of spatial transcriptomes calculated by the “AddModuleScore” function in Seurat (v4.3.0), we defined the immune cell aggregation index including two metrics: cell density and aggregation area. For the sake of comparability, all tissue aggregation indices were calculated at a scale of bin20. First, we calculated the immune cell gene set scores for each bin. Bins with score <0 were removed, then the bottom 5% bins were also removed, the retained bins were used for the subsequent analysis. Then, the same cell‐cell distance was calculated using an average distance of its eight nearest same‐cell neighbors. For example, considering N plasma cells in spatial slices, we calculated the distance for each plasma cell to its closest eight neighboring plasma cells, taking the average as the distance of that plasma cell to other nearby plasma cells. Finally, we got N distances representing the proximity of each plasma cell to its neighboring same cells. This distance measures discrete characteristics of same‐cell in the slice, known as cell density. Furthermore, to quantify same‐cell aggregation area and proportion, we constructed an undirected graph representing a specific type of immune cells, where each node in the graph corresponds to this type of cell within a spatial slice. Utilizing the Breadth‐First Search (BFS) algorithm, we identified all connected components in the undirected graph. Connected components with a minimum of two nodes were retained, and the total number of nodes within each connected component was called aggregation area. The overall percentage of all nodes was also calculated, known as aggregation proportion. If the aggregation area was greater than 500, we inferred that the immune cells were significantly clustered.

### Differential analysis and pathway enrichment analysis

The “FindMarkers” function in Seurat was implemented to identify differential expression across cell types or cell subtypes of interest by comparing average transcript levels in a given group cells with another group cells. For downstream analysis, genes satisfied a threshold logfc. threshold = 0.5 and a Bonferroni adjustment of *p‐*value < 0.05 as a statistical significance threshold. We engaged the R package clusterProfiler (v3.12.0) to perform Gene Ontology and pathways enrichment analysis for each group of DEGs.

### Scoring of signature gene sets

The gene signature scores of individual cells or spatial transcriptomics data set were calculated using the “AddModuleScore” function in seurat (v4.6.0) with default parameters. Immune cell‐related genes and keys pathway genes were shown in Table S4.

### Calculation of metabolic pathway activity and statistical analysis

The scMetabolism package (https://www.github.com/wu-yc/scMetabolism) was used to analyze the pathway activity at the single‐cell level in a high‐quality metabolic gene set [68]. In our study, a subset of gut epithelium cells was extracted from the Seurat object to compare changes in metabolic pathway activity in the absence of microbiota. We used the VISION quantification method in this package to evaluate the metabolic activity of each cell and finally obtained the activity score of gut epithelium cells in each metabolic pathway. For all the original scores, we normalized them to 0–1 at each pathway on GF and SPF, respectively. The normalization method was (V_cur_ − V_min_)/(V_max_ − V_min_), where Vmin was the minimum activity score and Vmax was the maximum activity score for one pathway. For all scMetabolism scaled score comparisons, statistical analyses were completed using Wilcoxon tests.

### Quantification and analysis of BAs

Targeted metabolomics was performed to measure BA (BA) metabolite concentrations in liver, cecum content, feces, and plasma samples from GF and SPF mice. A total of 36 BAs were prepared and quantified using liquid chromatography‐tandem mass spectrometry (LC‐MS/MS) in multi reaction monitoring (MRM) mode [69]. The liquid‐mass spectrometry system consisted of an ultra‐high performance liquid chromatography (Agilent UHPLC 1290, USA) and triple Quad mass spectrometer (Agilent QQQ‐MS 6500, USA). The BA metabolites were separated using an ACQUITY UPLC BEH C18 column (2.1 × 100 mm, 130 Å, 1.7 µm) and gradient elution with 0.01% formic acid in water and 0.01% formic acid in acetonitrile: methanol (70:30). The MultiQuant software was used for automatic identification and integration of MRM transitions (both positive and negative ions), with manual checks and default parameters. The acquired data was then analyzed in MassHunter Workstation Software (Agilent, USA) for peak integration, calibration, and quantification of individual BAs.

The calculation for BA content is given by the formula: A ∗ V/M, where A represents the concentration value obtained by substituting the integrated peak area of the target index in the sample into the standard curve (nmol/L), V denotes the extraction volume (L), M stands for the actual weighed mass of the sample (g), and the unit of measurement is nM/g.

The total BA levels for each sample were determined by accumulating all tested BAs. The significant differences in BA content between GF and SPF groups were identified using the wilcox.test method.

### Liver zonation and deconvolution

Stereo‐seq bins location along the lobule axis were inferred from the expression of a panel of reported landmark genes. For this study, we annotated zones by assigning bins (bin20) into nine layers based on the zonation score of a classic set of three pericentral (*Glul, Cyp2e1, Cyp1a2*) and four periportal (*Alb, Ass1, Asl, Cyp2f2*) landmark genes [70] based on the latest algorithm research [27]. For each bin, we calculated the module scores for central and portal landmark genes (CV score and PV score), respectively. Zonation score for each bin was defined as “PV score − CV score”. We then divided spatial bins into nine equal parts by bin numbers based on the increasingly ordered zonation scores and assigned them to nine discrete layers (layer 1–9) per spatial slide.

To identify differential gene expression in hepatocytes between GF and SPF mice, we integrated public single‐cell and single‐nucleus RNA‐seq data from healthy murine livers with our study's liver scRNA‐seq data [71]. We used Cell2Location for spatial data deconvolution and selected the maximum value per spot to determine the primary cell type. The Seurat “FindMarkers” function was applied to find DEGs across peri‐portal, mid‐portal, and central vein regions, comparing GF and SPF transcript levels. DEGs were filtered with a log fold change threshold of 0.25 and a Bonferroni‐adjusted *p‐*value < 0.05. Metascape (http://metascape.org) was used for Gene Ontology and pathway enrichment analysis of the DEGs.

### Association of human GWAS and genetic disease data of intestinal epithelial cells cell types

To evaluate the enrichment of DEGs intestinal epithelial cells of GF and SPF mice associated with human diseases or physiological phenotypes, we performed a comprehensive gene analysis across all phenotypes in the GWAS catalog. The enrichment of differentially expressed genes in GWAS traits was determined using Fisher's test (Table S8).

### Immunofluorescence staining and confocal imaging

The ileal portion of the small intestine was excised and tissues were snap‐frozen in precooled TissueTek OCT on dry ice, then stored at −80°C. 10 µm tissue sections were cut on a RM2235 cryostat (Leica, Germany) and adhered to Superfrost Plus slides (VWR, USA). The slides were placed in a 3% paraformaldehyde solution for fixation for 2 h, followed by antigen retrieval in EDTA antigen retrieval buffer. Subsequently, endogenous peroxidase activity was quenched using 3% hydrogen peroxide, and serum blocking was performed with bovine serum albumin (BSA). The primary antibody was incubated overnight at 4°C, after which the slides were washed with PBS and incubated with the secondary antibody at 37°C for 30 min. Nuclear staining was carried out using DAPI, with incubation for 10 min at room temperature in the dark. The following antibodies were used for staining: anti‐Perilipin (Abcam, ab52356, UK) and anti‐LIPA (Proteintech, 12956‐1‐AP, USA). After staining, the slides were examined under a NEXcope confocal microscope, and the images were processed and analyzed using ImageJ software.

## AUTHOR CONTRIBUTIONS

**Juan Shen**: Project design; tissue collection; investigation; data analysis and interpretation; code and data curation; writing—original draft; writing—review and editing. **Weiming Liang**: Data analysis; code and data curation; investigation. **Ruizhen Zhao**: Project design; tissue collection; visualization. **Yang Chen**: Data interpretation of intestinal mucosa; writing—original draft of intestinal mucosa. **Yanmin Liu**: Bile acid data analysis and interpretation; writing—original draft of bile acid; writing—review and editing; **Wei Chen**: Germ‐free mouse husbandry; tissue dissection; experiments. **Tailiang Chai**: Project design; tissue collection. **Yin Zhang**: Project design; tissue collection. **Silian Chen**: Image editing; data curation. **Jiazhe Liu**: Code curation. Xueting Chen: Code curation. **Yusheng Deng**: Visualization. **Zhao Zhang**: Visualization. **Yufen Huang**: 16S data analysis, **Huanjie Yang**: Visualization. **Li Pang**: Experiments. **Qinwei Qiu**: Visualization. **Haohao Deng**: Experiments. **Shanshan Pan**: Experiments. **Linying Wang**: Experiments. **Jingjing Ye**: Experiments. **Wen Luo**: Visualization. **Xuanting Jiang**: Project design. **Xiao Huang**: Experiments. **Wanshun Li**: Experiments. **Lixian Liang**: Experiments. **Lu Zhang**: Writing—review. **Li Huang**: Writing—review. **Zhimin Yang**: Writing—review. Rouxi Chen: Resources. **Junpu Mei**: Resources. **Zhen Yue**: Resources. **Hong Wei**: Conceptualization; project design; resources. **Karsten Kristiansen**: writing—original draft; writing—review and editing. **Lijuan Han**: Conceptualization; project design; writing—review. **Xiaodong Fang**: Conceptualization; project design; investigation; resources; writing—review and editing; supervision; funding acquisition.

## CONFLICT OF INTEREST STATEMENT

The authors declare no conflicts of interest.

## ETHICS STATEMENT

The study protocol was approved by the central ethics committee at Huazhong Agricultural University and the institutional review board of BGI ethical clearance (ethics number: BGI‐IRB A21028‐T2). All experimental methods in this study were performed following the guide of the Three Rs principle.

## Supporting information


**Figure S1.** Fecal microbiota composition in specific pathogen‐free (SPF) mice.
**Figure S2.** Global quality summary of single‐cell RNA sequencing (scRNA‐seq) data.
**Figure S3.** Global heatmap profiling of immune and gut epithelial cells.
**Figure S4.** Visualization depicting the spatial distribution of cells and the expression of key genes.
**Figure S5.** B cell subtypes and molecular characteristics, as well as heterogeneity in tissue distribution.
**Figure S6.** Spatial distribution and heterogeneity of plasma cell subtypes across tissues, including markers *Igha, Ighg1, Ighg2b, Ighg2c,* and *Ighg3*.
**Figure S7.** Myeloid cell subtypes annotation, tissue heterogeneity, the number of differentially expressed genes (DEGs), and macrophage subtype enrichment result.
**Figure S8.** Neutrophil functions at different developmental stages between germ‐free (GF) and SPF.
**Figure S9.** Natural killer (NK)/T cell subtypes annotation, tissue heterogeneity, and the number of DEGs.
**Figure S10.** Gene Ontology (GO) enrichment analysis results of the top NK/T subtypes with the highest number of DEGs and the homing process of intestinal intraepithelial T cells.
**Figure S11.** Heatmaps depicting the impact of microbial depletion on gene expression across lymphoid subsets from six immunological gene lists in ImmPort.
**Figure S12.** Integrative clustering and annotation of the intestine.
**Figure S13.** The detailed landscape of lipid absorption and metabolism in ileal epithelial cells is significantly altered following microbial depletion, including chylomicron formation, storage, lipolysis, maturation, and transport.
**Figure S14.** Functional enrichment analysis of DEGs and altered lipid metabolism process in the liver.
**Figure S15.** BA tissue distribution and hepatic zonation of metabolism‐related genes in GF and SPF mice.
**Figure S16.** Integrated analysis revealing perturbed bile ducts in GF mice.

**Table S1.** The guaranteed nutrient content per kilogram for germ‐free (GF) and specific pathogen‐free (SPF) mice.
**Table S2.** Baseline physiological and biochemical characteristics between eleven male 10‐week‐old GF and ten male 10‐week‐old SPF mice.
**Table S3.** Comprehensive overview of analyzed samples and experimental platforms.
**Table S4.** The gene sets related to immune cells that were utilized for calculating scores through the “AddModuleScore” function of Seurat.
**Table S5.** Gene Ontology (GO) enrichment analysis of differentially expressed genes across lymphoid cell subtypes in our multi‐organ database.
**Table S6.** Go enrichment analysis of differentially expressed genes across intestinal epithelial subtypes.
**Table S7.** Genes associated with nutrient absorption, antibacterial defense, antiviral responses, virus receptors, MHCII molecules, mucosal barrier integrity, chemokine signaling, and pattern recognition receptors were employed to investigate heterogeneity in gut epithelial function.
**Table S8.** Association of differentially expressed genes of intestinal epithelium with common human traits and genetic diseases.
**Table S9.** Lipid‐related genes involving in triacylglycerol synthesis, fatty acid oxidation, lipid droplet formation, and lipolysis in hepatocytes and ileal epithelium, as well as fatty acid uptake, chylomicron assembly, transport, and secretion in ileal epithelium.
**Table S10.** Bile acid‐related genes and their corresponding functions, including synthesis, transport, reabsorption, conjugation, and receptor.

## Data Availability

The raw matrices of livers' scRNA‐seq data are deposited in https://db.cngb.org/search/project/CNP0003996/. The raw matrices of spleens' scRNA‐seq data are deposited in https://db.cngb.org/search/project/CNP0003930/. The raw matrices of scRNA‐seq data of blood, bone marrow, ileum, cecum, colon, and thymus are deposited in https://db.cngb.org/search/project/CNP0005580/. All the scRNA‐seq rds files are deposited in https://db.cngb.org/search/project/CNP0005580/. The raw matrices of livers' Stereo‐seq data are deposited in https://db.cngb.org/stomics/project/STT0000034/spatialGeneExpression. The raw matrices of spleens' Stereo‐seq data are deposited in https://db.cngb.org/stomics/project/STT0000089/spatialGeneExpression. The raw matrices of Stereo‐seq data of ileum, cecum, colon, and thymus are deposited in https://db.cngb.org/stomics/project/STT0000129/spatialGeneExpression. All the Stereo‐seq rds files are deposited in https://db.cngb.org/stomics/project/STT0000129/spatialGeneExpression. Computer code used for processing the scRNA‐seq and Stereo‐seq data analysis is available at github link: https://github.com/BGI-Intestines/Germ-free-mice. Supplementary materials (figures, tables, scripts, graphical abstract, slides, videos, Chinese translated version, and updated materials) may be found in the online DOI or iMeta Science http://www.imeta.science/.
