# Correction to “Cross‐tissue multi‐omics analyses reveal the gut microbiota's absence impacts organ morphology, immune homeostasis, bile acid and lipid metabolism”

**DOI:** 10.1002/imt2.70092

**Published:** 2025-11-16

Shen, Juan, Weiming Liang, Ruizhen Zhao, Yang Chen, Yanmin Liu, Wei Cheng, Tailiang Chai, et al. 2025. “Cross‐tissue multi‐omics analyses reveal the gut microbiota's absence impacts organ morphology, immune homeostasis, bile acid and lipid metabolism.” *iMeta* 4, e272. https://doi.org/10.1002/imt2.272.

In the author list of the main text and the supplementary materials, the corresponding author's name “**Kristiansen Karsten**” should be written as “**Karsten Kristiansen**.”

We apologize for this error.

