# Supplementary material for: Cross‐tissue multi‐omics analyses reveal the gut microbiota's absence impacts organ morphology, immune homeostasis, bile acid and lipid metabolism
Source: Imeta. 2025 Feb 14;4(1):e272. doi: 10.1002/imt2.272 (PMC11865341; doi:10.1002/imt2.272)
Supplement: Supplementary file 1 — Figure S1. Fecal microbiota composition in specific pathogen‐free (SPF) mice. Figure S2. Global quality summary of single‐cell RNA sequencing (scRNA‐seq) data. Figure S3. Global heatmap profiling of immune and gut epithelial cells. Figure S4. Visualization depicting the spatial distribution of cells and the expression of key genes. Figure S5. B cell subtypes and molecular characteristics, as well as heterogeneity in tissue distribution. Figure S6. Spatial distribution and heterogeneity of plasma cell subtypes across tissues, including markers Igha, Ighg1, Ighg2b, Ighg2c, and Ighg3. Figure S7. Myeloid cell subtypes annotation, tissue heterogeneity, the number of differentially expressed genes (DEGs), and macrophage subtype enrichment result. Figure S8. Neutrophil functions at different developmental stages between germ‐free (GF) and SPF. Figure S9. Natural killer (NK)/T cell subtypes annotation, tissue heterogeneity, and the number of DEGs. Figure S10. Gene Ontology (GO) enrichment analysis results of the top NK/T subtypes with the highest number of DEGs and the homing process of intestinal intraepithelial T cells. Figure S11. Heatmaps depicting the impact of microbial depletion on gene expression across lymphoid subsets from six immunological gene lists in ImmPort. Figure S12. Integrative clustering and annotation of the intestine. Figure S13. The detailed landscape of lipid absorption and metabolism in ileal epithelial cells is significantly altered following microbial depletion, including chylomicron formation, storage, lipolysis, maturation, and transport. Figure S14. Functional enrichment analysis of DEGs and altered lipid metabolism process in the liver. Figure S15. BA tissue distribution and hepatic zonation of metabolism‐related genes in GF and SPF mice. Figure S16. Integrated analysis revealing perturbed bile ducts in GF mice. [file IMT2-4-e272-s002.docx]

**Supporting information to**

**Cross-tissue multi-omics analyses reveal the gut microbiota’s absence impacts organ morphology, immune homeostasis, bile acid and lipid metabolism**

**Running title:** Cross-tissue multi-omics study of germ-free and specific pathogen-free mice

Juan Shen^1,2#^, Weiming Liang^1#^, Ruizhen Zhao^1#^, Yang Chen^3#^, Yanmin Liu^3#^, Wei Cheng^4#^, Tailiang Chai^1^, Yin Zhang^1^, Silian Chen^1^, Jiazhe Liu^1^, Xueting Chen^1^, Yusheng Deng^3^, Zhao Zhang^1^, Yufen Huang^1^, Huanjie Yang^1^, Li Pang^1^, Qinwei Qiu^3^, Haohao Deng^5^, Shanshan Pan^6^, Linying Wang^1^, Jingjing Ye^1^, Wen Luo^7,8^, Xuanting Jiang^7,8^, Xiao Huang^1^, Wanshun Li^1^, Elaine Lai-Han Leung^8,9,10^, Lu Zhang^11^, Li Huang^3^, Zhimin Yang^3^, Rouxi Chen^12^, Junpu Mei^1,12^, Zhen Yue^12^, Hong Wei^4,13*^, Kristiansen Karsten ^1,14*^, Lijuan Han^7,8,15*^, Xiaodong Fang^3,12*^

^1^ BGI Research, Shenzhen, China

^2^ Qingdao-Europe Advanced Institute for Life Sciences, BGI Research, Qingdao, China

^3^ State Key Laboratory of Traditional Chinese Medicine Syndrome, State Key Laboratory of Dampness Syndrome of Chinese Medicine Syndrome, The Second Affiliated Hospital of Guangzhou University of Chinese Medicine, Guangzhou, China

^4^ College of Animal Sciences and Technology, Huazhong Agricultural University, Wuhan, China

^5^ BGI Research, Beijing, China

^6^ BGI Research, Qingdao, China

^7^ Kangmeihuada (KMHD) GeneTech Co., Ltd., Shenzhen, China

^8^ Zhuhai UM Science & Technology Research Institute-Kangmeihuada (KMHD) joint lab, Zhuhai, China

^9^ Cancer Center, Faculty of Health Sciences, University of Macau, Macau (SAR), China

^10^ MOE Frontiers Science Center for Precision Oncology, University of Macau, Macau (SAR), China

^11^ Department of Computer Science, Hong Kong Baptist University, Hong Kong, China

^12^ BGI Research, Sanya, China

^13^ Yu‐Yue Pathology Scientific Research Center, Chongqing, China

^14^ Laboratory of Genomics and Molecular Biomedicine, Department of Biology, University of Copenhagen, Copenhagen, Denmark

^15^ Kangmei Pharmaceutical Co., Ltd., Jieyang, China

^#^ These authors contributed equally: Juan Shen, Weiming Liang, Ruizhen Zhao, Yang Chen, Yanmin Liu, Wei Cheng

^*^ Correspondence: [fangxd@genomics.cn](mailto:fangxd@genomics.cn) (Xiaodong Fang), [hlj@kmhdgene.com](mailto:hlj@kmhdgene.com) (Lijuan Han), [kk@bio.ku.dk](mailto:kk@bio.ku.dk) (Kristiansen Karsten), [weihong63528@163.com](mailto:weihong63528@163.com) (Hong Wei)


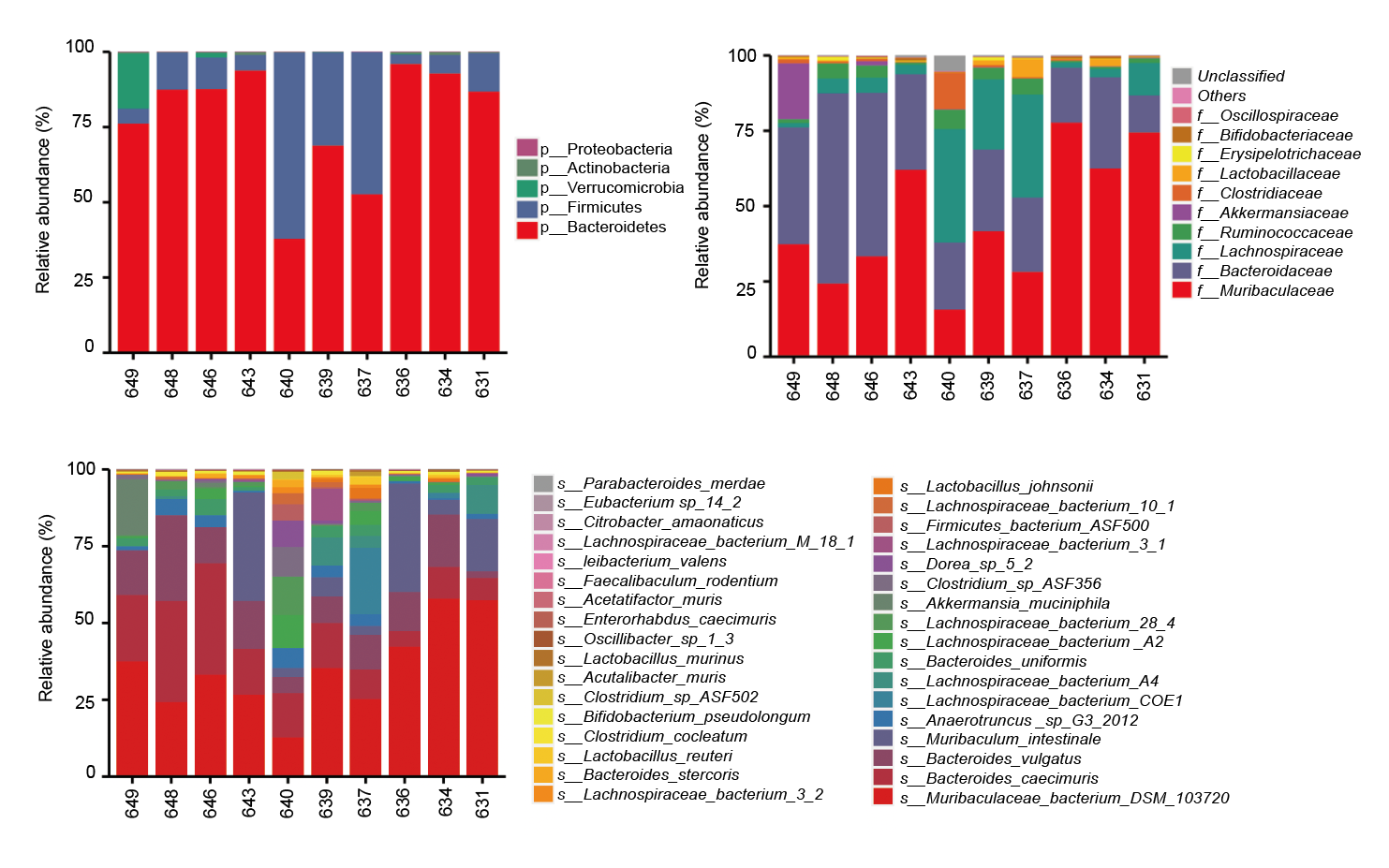


**Figure S1** **Fecal microbiota composition in specific pathogen-free (SPF) mice.**


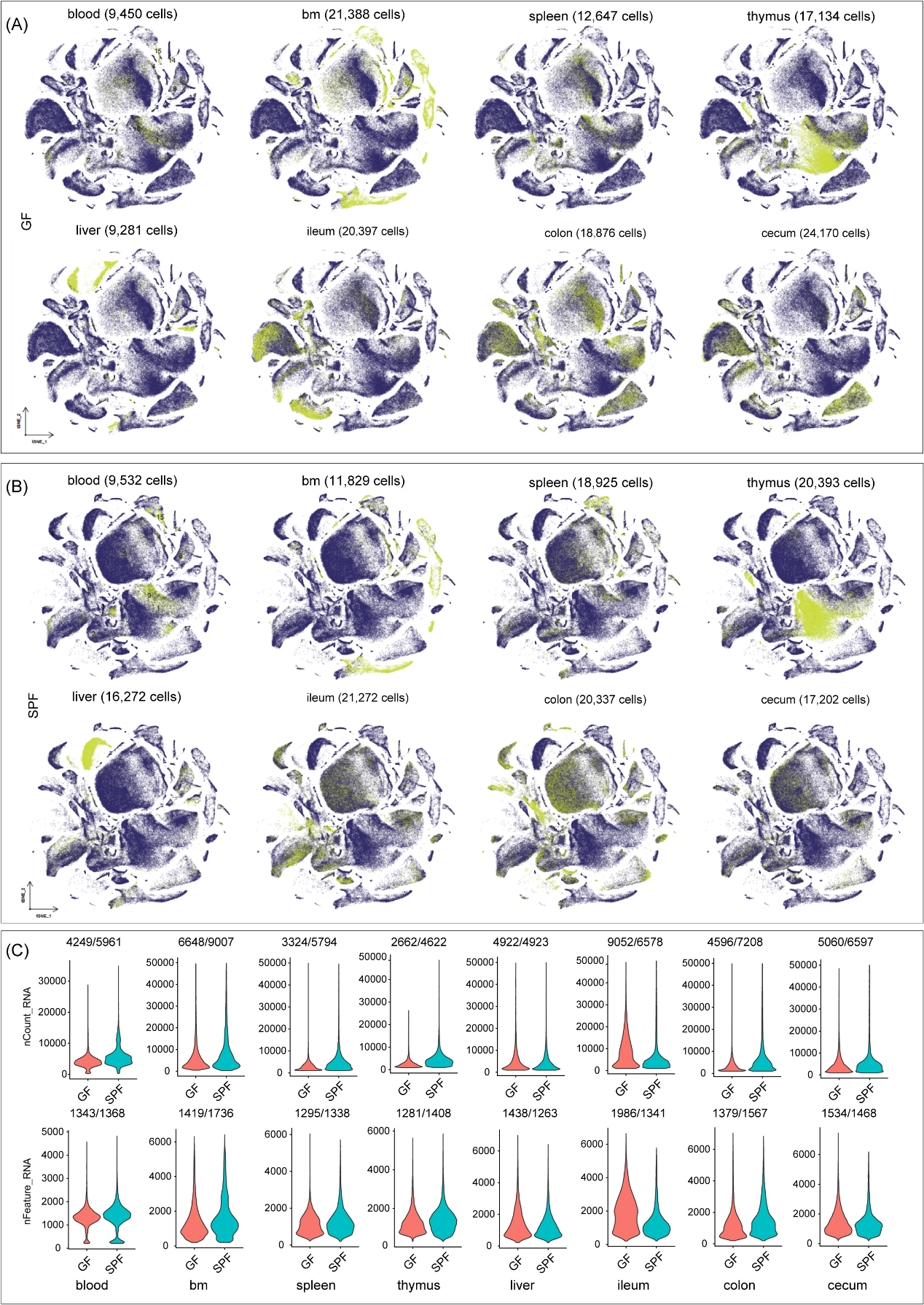


**Figure S2** Global quality summary of single-cell RNA sequencing (scRNA-seq) data**.** (A-B) TSNE projection of global clustering was employed to display the cell numbers profiled between GF mice and SPF mice. (C) After strict quality control (QC) of scRNA-seq data, the distribution of UMI counts and gene counts between GF and SPF mice are displayed.


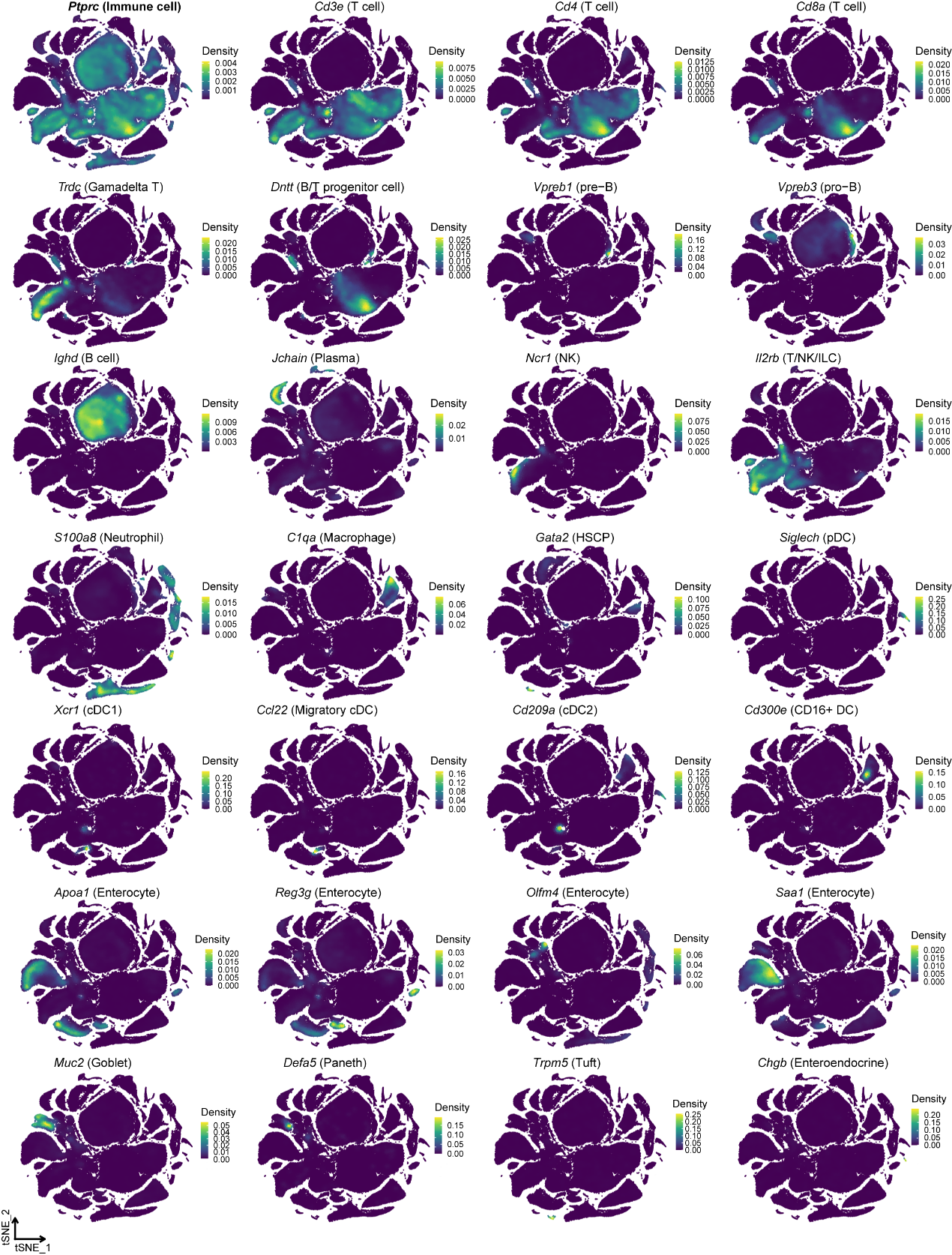


**Figure S3** Global **heatmap** profiling of immune cells and gut epithelium cells**.** The selected canonical markers for different levels of immune cell (*Ptprc*) annotation include T cell (*Cd3e*, *Cd4* and *Cd8a*), gamma-delta T cell (*Trdc*), progenitor cell of B or T (*Dntt*), pre-B (*Vpreb1*), pro-B (*Vpreb3*), B cell (*Ighd*), plasma cell (*Jchain*), NK cell (*Ncr1*), neutrophil cell (*S100a8*), macrophage cell (*C1qa*), HSCP (*Gata2*), pDC (*Siglech*), cDC1 (*Xcr1*), cDC2 (*Cd209a*), CD16^+^ DC (*Cd300e*), migratory DC (*Ccl22*), tip-villus epithelial cell (*Apoa1* and *Saa1*), mid-villus epithelial cell (*Reg3g* and *Retnlb*), stem cell region (*Olfm4*), goblet cell (*Muc2*), tuft cell (*Trpm5*), enteroendocrine cell (*Chgb*) and paneth cell (*Defa5*).


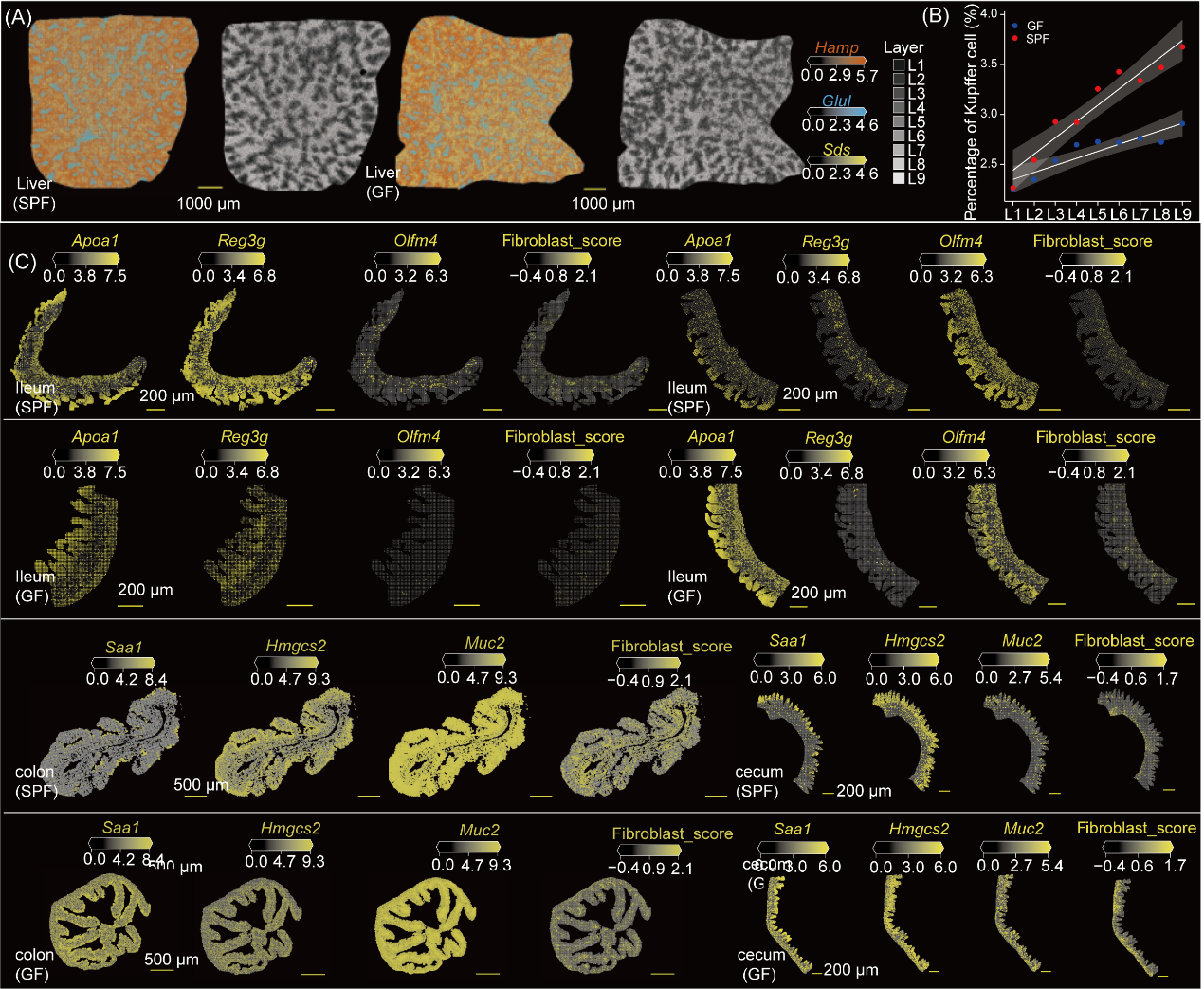


**Figure S4** **Visualization depicting the spatial distribution of cells and the expression of key genes.** We selected genes or cells that represent the physiological functional structure of the tissue to describe the effect at the level of the spatial transcriptome. (A) For the liver zonation, marker genes representing pericentral (*Glul*), middle (*Hamp*) and periportal (*Sds*) hepatocytes are shown. Spatial visualization of the nine zonation layers spanning from central vein to portal vein within Stereo-seq sections. (B) The proportion of Kupffer cell in each zonation layer of Stereo-seq section. (C) For the gut spatial stratification, we primarily focused on the epithelial apex of ileum (*Apoa1*) and colon (*Saa1* and *Hmgcs2*), the antimicrobial niche region of ileum (*Reg3g*), the stem cell region at the bottom of ileal epithelium (*Olfm4*), the goblet cell of colon (*Muc2*), and fibroblasts.

**
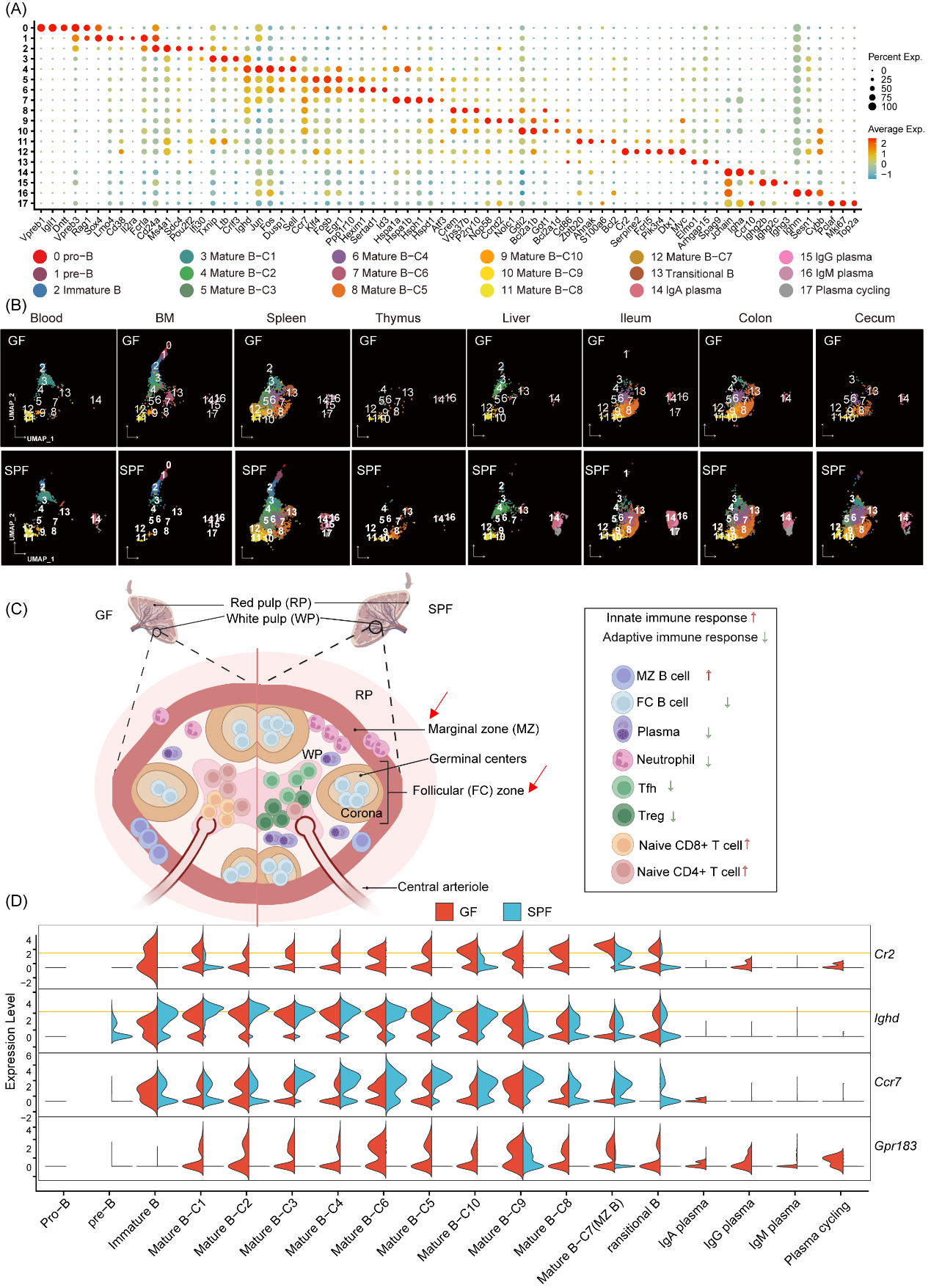
**

**Figure S5** **B cell subtypes and molecular characteristics, as well as heterogeneity in tissue distribution.** (A) Dot plot showing marker genes for B cells after integration of data from eight tissues. Dot size represents the cell proportion for each cluster expressing the given marker gene. The color indicates the relative log-normalized gene expression level for a given marker gene in each tissue. (B) UMAP visualization of the subtypes of B cell across eight tissues in GF or SPF mice are displayed. (C) Schematic diagram of splenic architecture, cellular localization, and proportional variations of GF and SPF mice. (D) Expression of four genes associated with B development in splenic B cell compartments. Compared to SPF mice, we found *Cr2* expression in MZ B cell of GF mice (Mature B-C7) had a significant up-regulation and *Ighd* expression had a significant down-regulation*.* For follicular B cells (Mature B-C1-C6, C8-C10), expression of *Cr2* gene in SPF mice was almost undetectable, which is consistent with a previous study. However, we observed that the expression of the *Cr2* gene in GF mice FC B cells increased significantly after microbial deletion. Gene *Ccr7* and *Gpr183* mediate chemotaxis and migration of splenic T cells and B cells, participating in the formation of splenic structures. The down-regulation of *Ccr7* expression and up-regulation of *Gpr183* in GF mice imply that the localization and proportion of T and B cells in the spleen are affected.

**
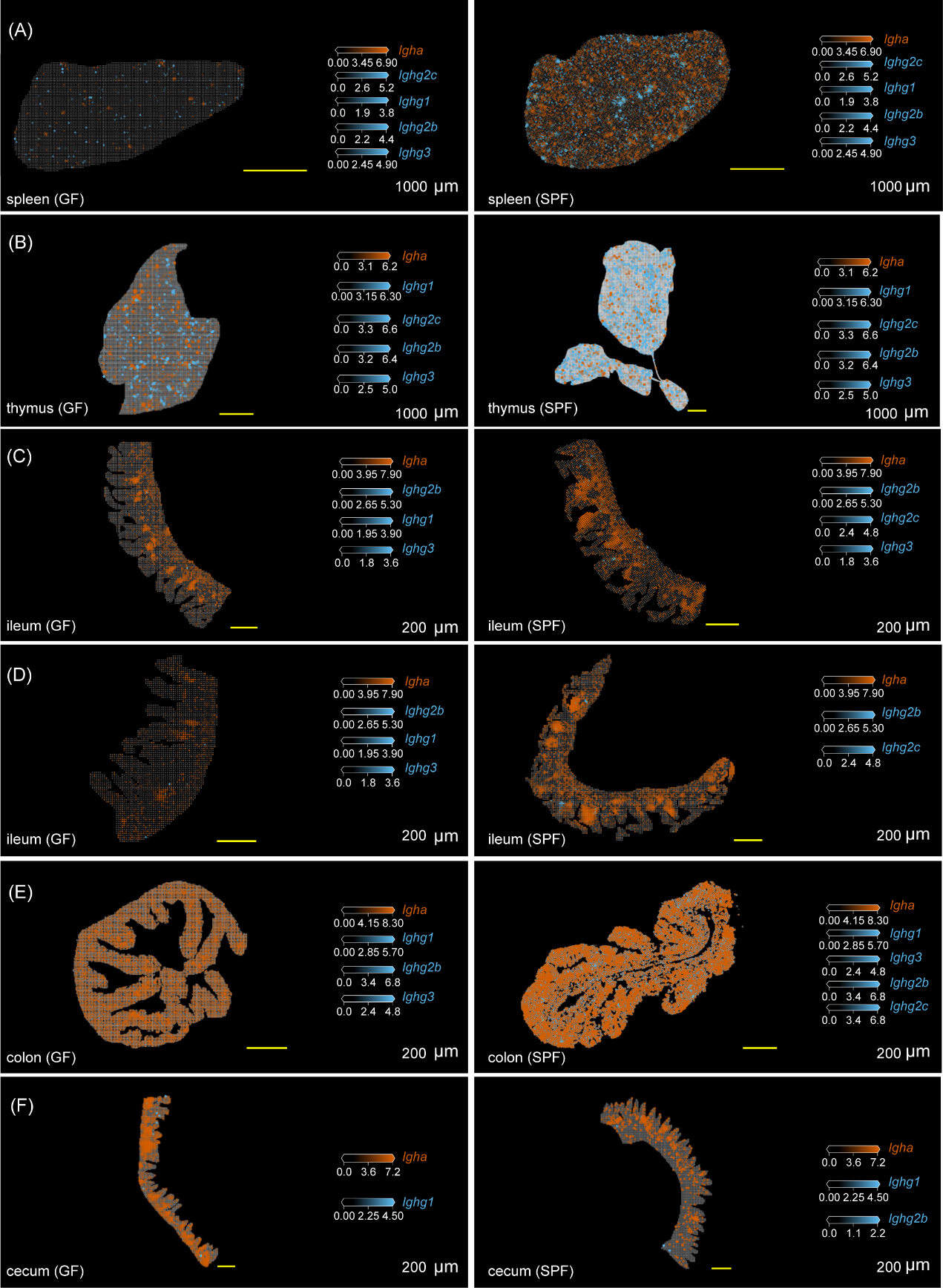
**

**Figure S6** Spatial distribution and heterogeneity of plasma cell subtypes across tissues, including markers *Igha*, *Ighg1*, *Ighg2b*, *Ighg2c*, and *Ighg3***.** The brighter the color, the higher the expression of the gene.

**
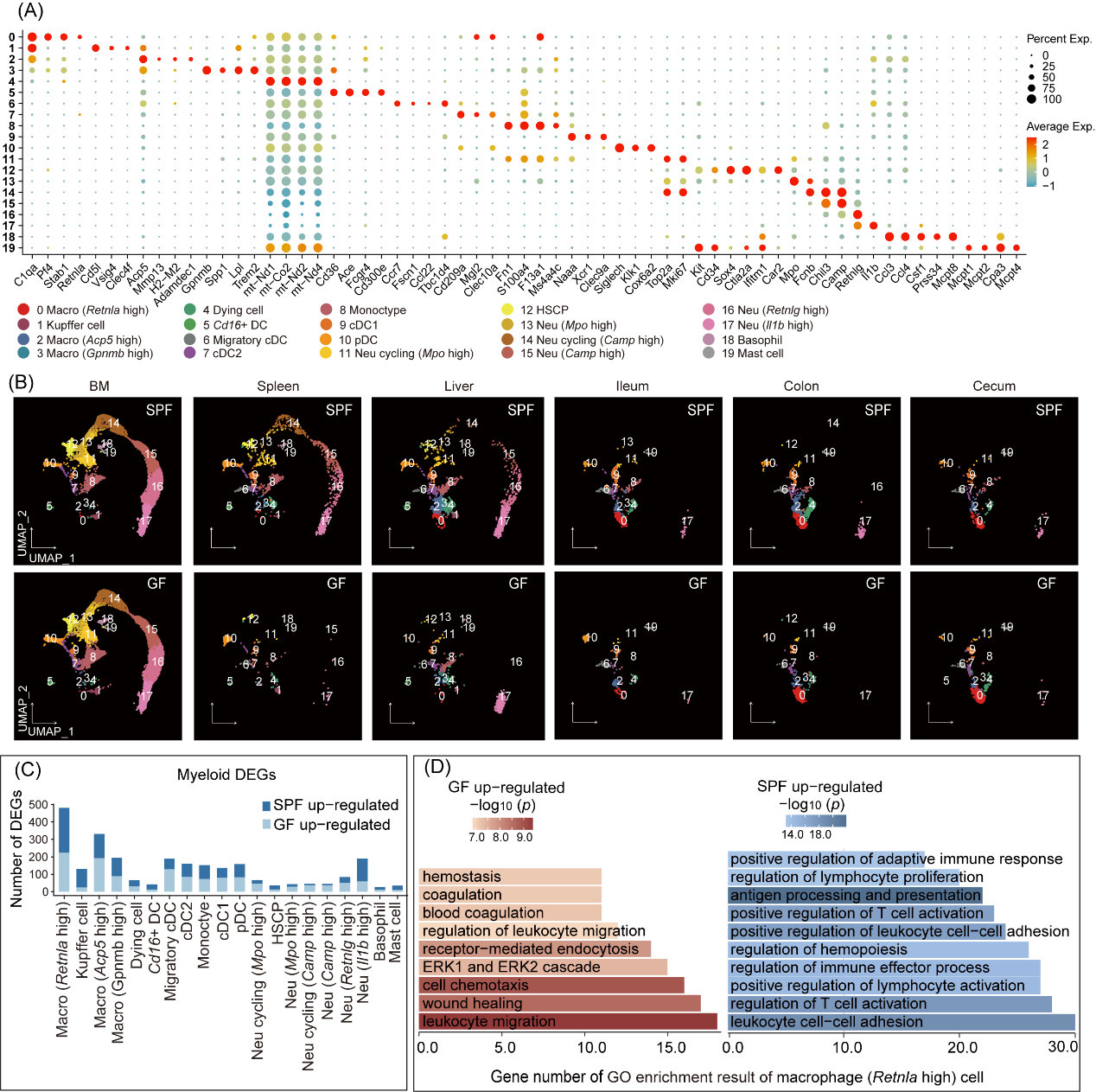
**

**Figure S7** **Myeloid cell subtypes annotation, tissue heterogeneity, the number of differentially expressed genes (DEGs) and macrophage subtype enrichment result.** (A) The selected marker genes for myeloid subtypes annotation. (B) UMAP visualization of the subtypes of myeloid cells across eight tissues in GF or SPF mice are displayed. (C) The number of DEGs of myeloid cell subtypes. (D) Gene Ontology (GO) enrichment analysis of macrophage in large intestine.


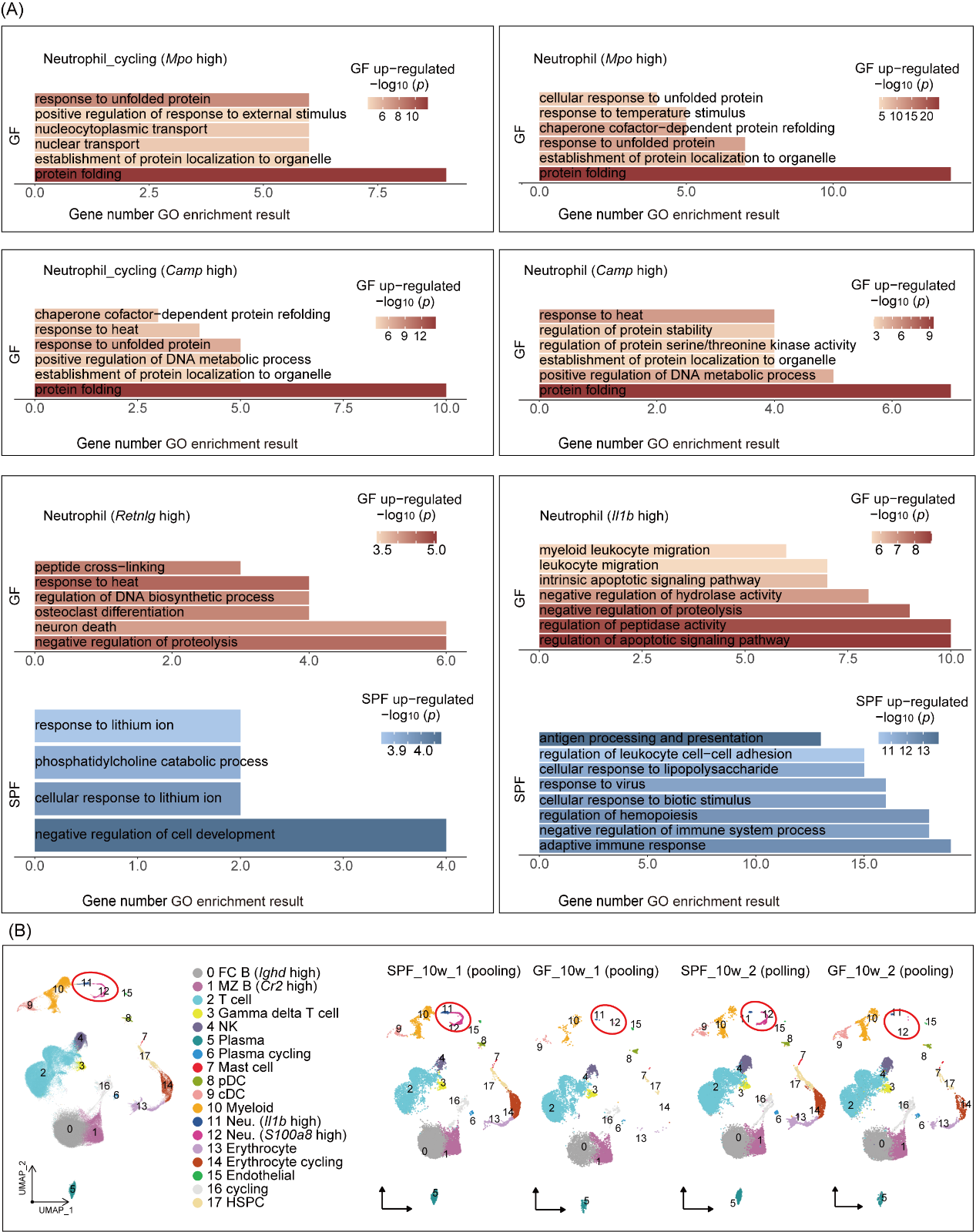


**Figure S8** **Neutrophil functions at different developmental stages between germ-free (GF) and SPF.** (A) Go enrichment analysis of neutrophils at different developmental stages. (B) UMAP distribution of neutrophils in the spleen of different batch.

**
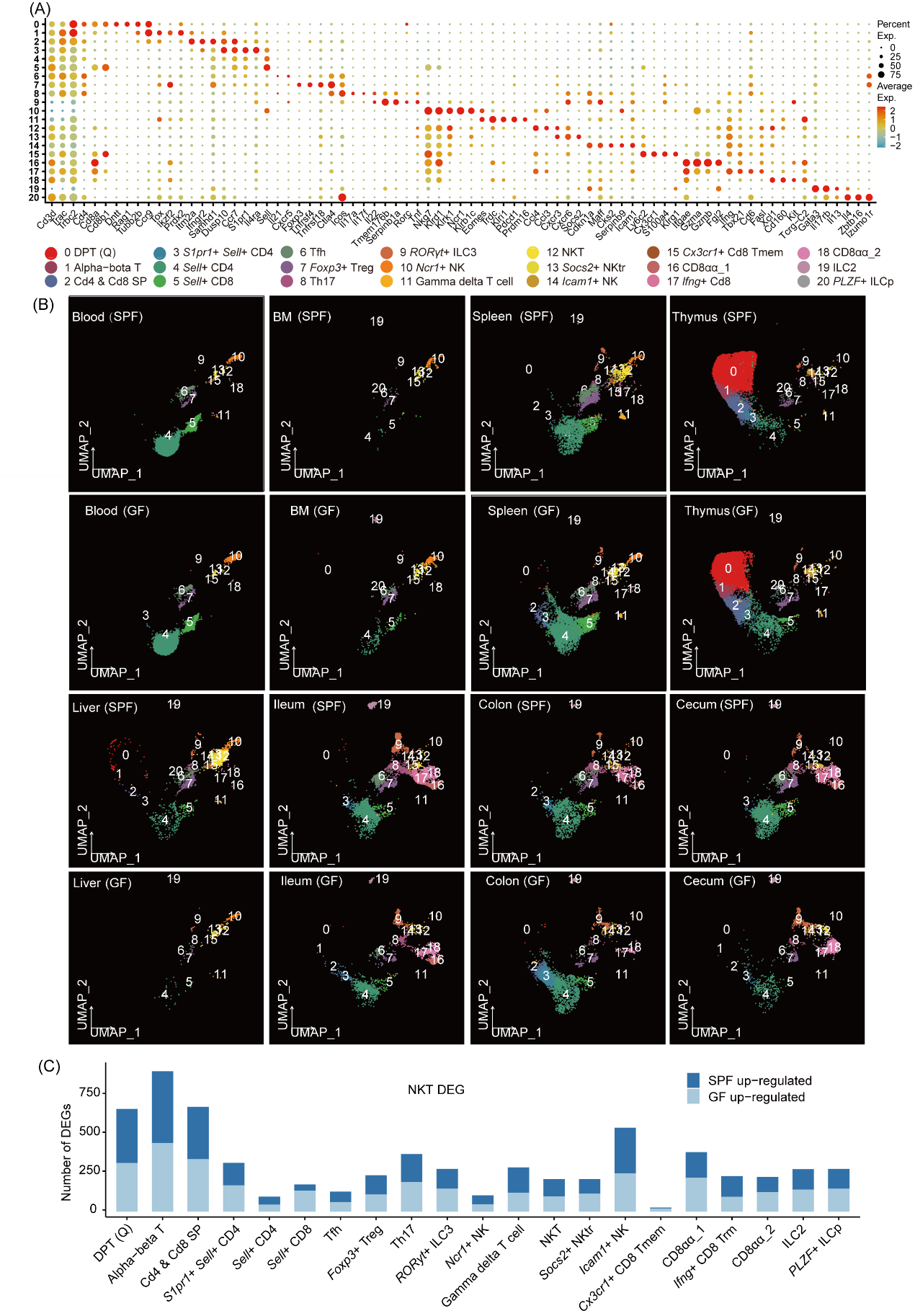
**

**Figure S9** **Natural killer (NK)/T cell subtypes annotation, tissue heterogeneity, and the number of DEGs.** (A) The selected marker genes for NK/T subtypes annotation. (B) UMAP visualization of the subtypes of NK/T cell across eight tissues in GF or SPF mice are displayed. (C) The count of DEGs across all NK/T cell subtypes.

**
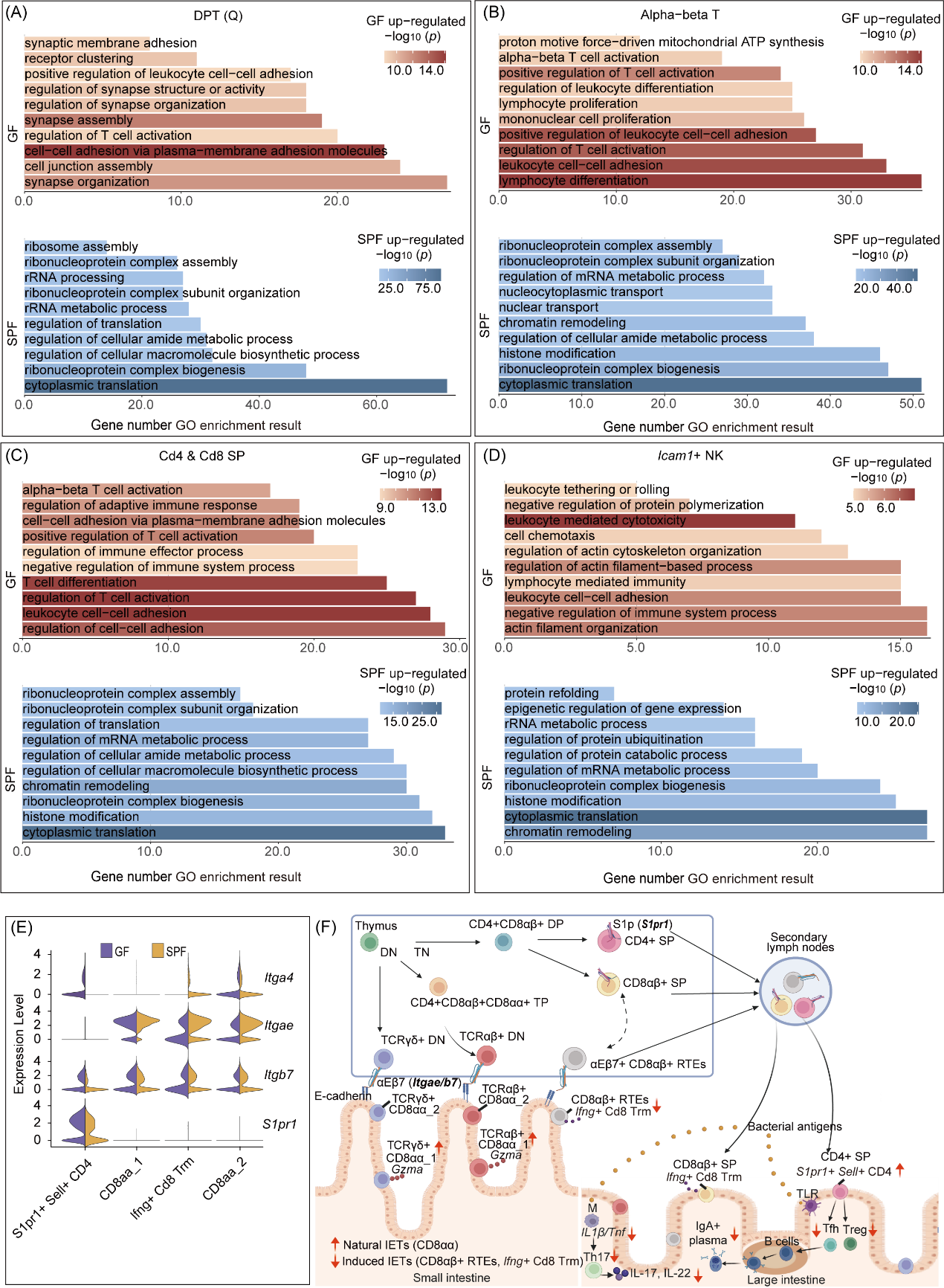
**

**Figure S10** **Gene Ontology (GO) enrichment analysis results of the top NK/T subtypes with the highest number of DEGs and the homing process of intestinal intraepithelial T cells.** (A-D) Go enrichment analysis for three early developmental state T cell subtypes and specific NK cell subtype in the thymus. (E) The expression of four key genes controlling T lymphocyte homing into different intestinal segments. (F) The migration pathway of T lymphocytes from the thymus to the intestines, as well as their alternation in the absence of microbes. Red arrows mark up- or down-regulation in GF mice.

**Figure S11** **Heatmaps depicting the impact of microbial depletion on gene expression across lymphoid subsets from six immunological gene lists in ImmPort.** The six immunologically relevant gene lists include antigen processing and presentation, antimicrobial genes, chemokines, cytokine and receptors, interferons TNF and TGF-beta family members gene, interleukins and receptors gene. We observed significant downregulation of genes involved in antigen processing and presentation, and genes encoding *H2-A*, *H2-E* and *Hsp70/90* mRNAs across most of subtypes, antimicrobial gene including *Slpi* in B/Plasma cells, *IL-17A* and *IL-22* in Th17 cells, *Ifng* in T/NK cells, *Isg15, Ccl5, Cxcl10* and *Ifih1* in T/NK/ILCs/macrophages, and *Il1b* in monocytes/macrophages.

**
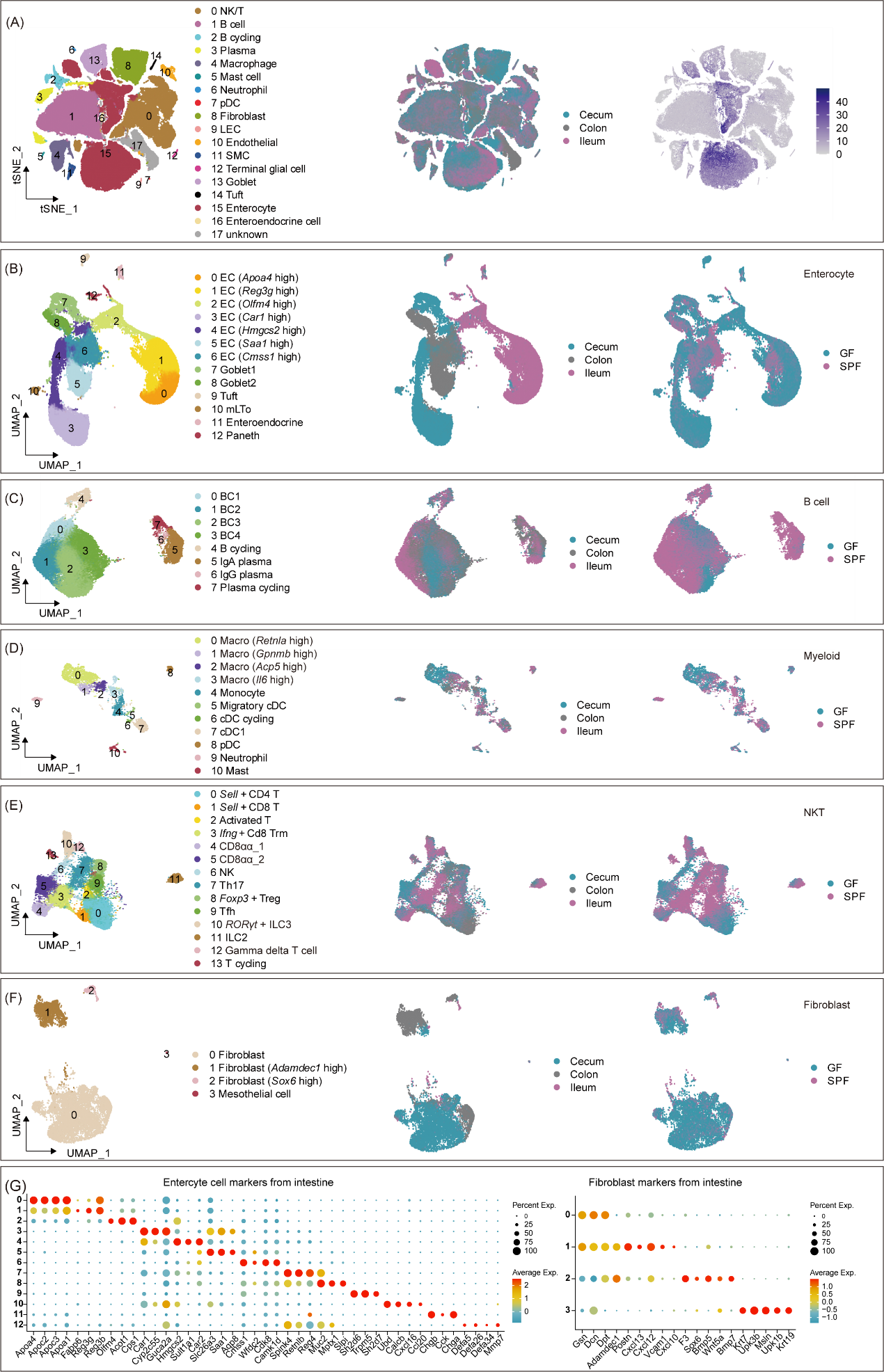
**

**Figure S12** **Integrative clustering and annotation of the intestine.** (A) UMAP plots were generated to visualize the 18 clusters of cells annotated in the ileum, colon, and cecum, demonstrating no apparent batch effect among the three intestinal segments. Additionally, a UMAP plot of the expression distribution of the percentage of mitochondrial genes was generated. (B-F) Similar UMAP plots were created for enterocyte cell subtypes (13 clusters, B), B cell subtypes (8 clusters, C), NK/T cell subtypes (14 clusters, D), myeloid cell subtypes (11 clusters, E), and fibroblast cell subtypes (4 clusters, F). All subtypes of the integrated intestine were subjected to deconvolution using Cell2location. (G) The selected marker genes for enterocyte cell subtypes and fibroblast cell subtypes annotation. These plots revealed no noticeable batch effect between different intestinal segments and between samples of GF and SPF mice.


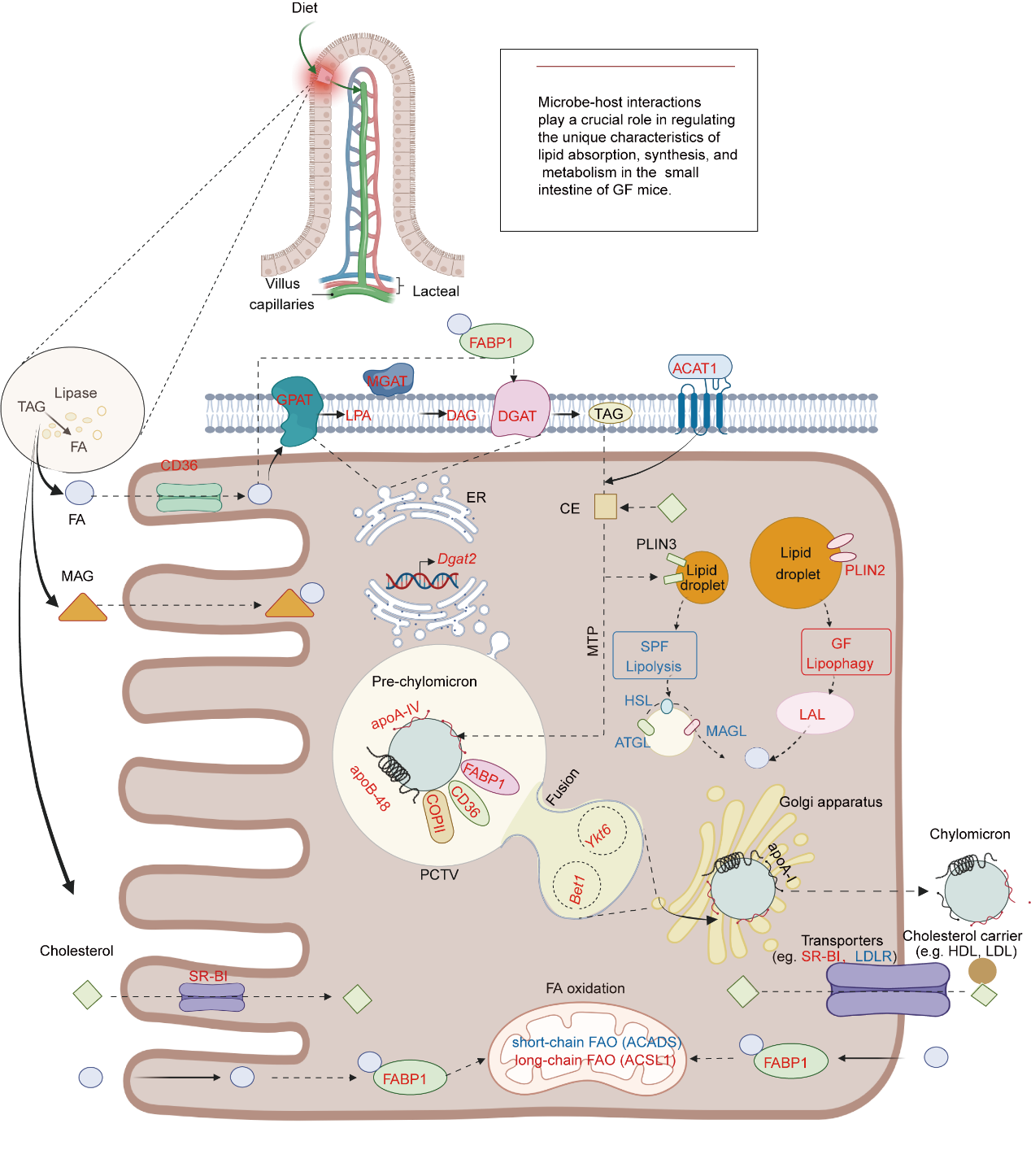
**Figure S13** **The detailed landscape of lipid absorption and metabolism in ileal epithelial cells is significantly altered following microbial depletion, including chylomicron formation, storage, lipolysis, maturation, and transport.** TAG, triacylglycerol; FA, fatty acid; MAG, monoacylglycerol; DAG, diacylglycerol; ER, endoplasmic reticulum; GPAT, glycerol-3-phosphate acyltransferase; LPA, lysophosphatidic acid; MGAT, monoacylglycerol acyltransferase; DGAT, diacylglycerol acyltransferases; CE, cholesteryl esters; MTP, microsomal triglyceride transfer protein; PCTV, pre-chylomicron transport vesicles; SR- BI, scavenger receptor class B type I; FAO, fatty acid oxidation; COPII, coat protein complex II.


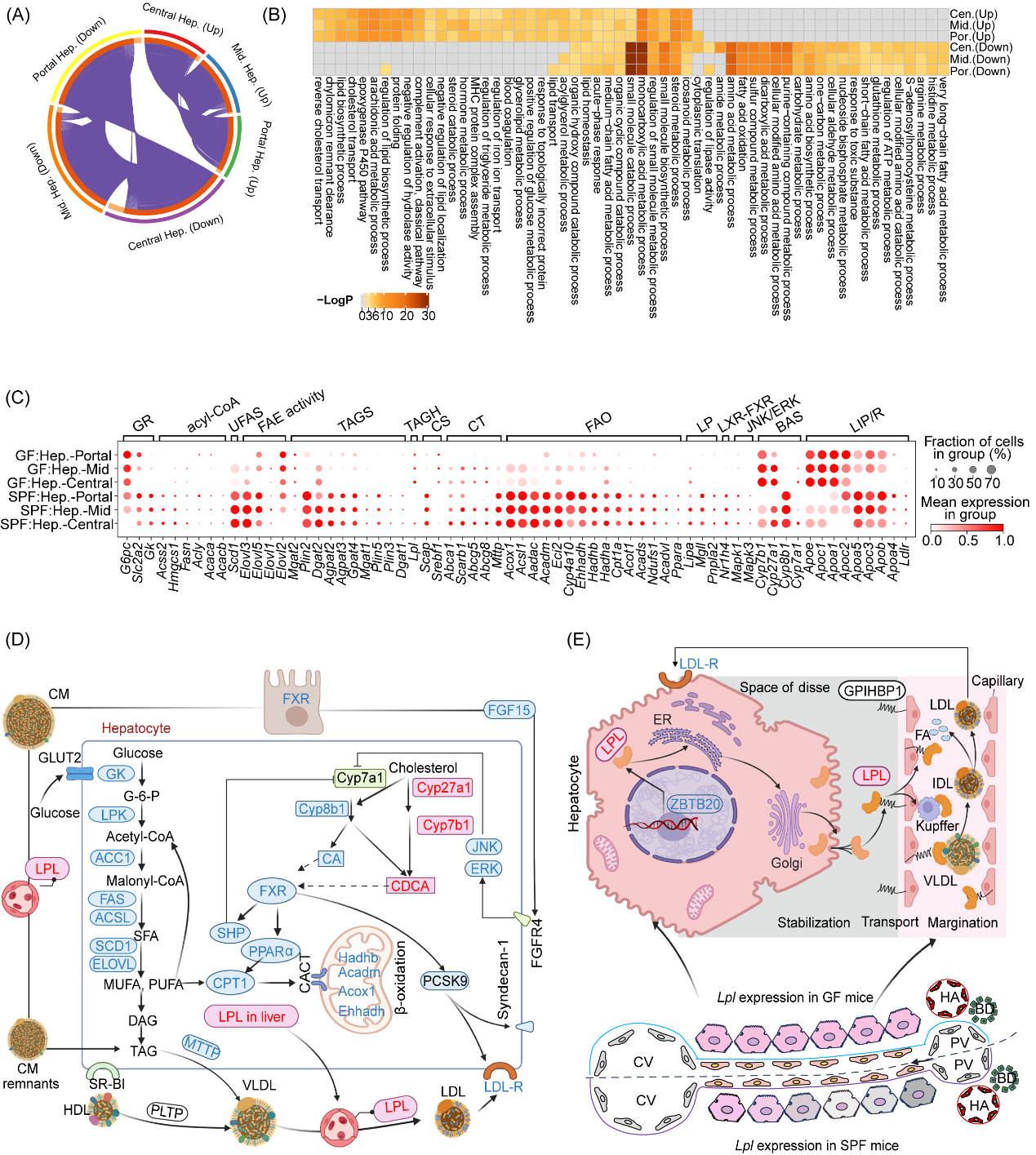


**Figure S14** **Functional enrichment analysis of DEGs and altered lipid metabolism process in the liver**. (A) Circos plot displaying the overlaps among the DEG lists (FC > 0.25) from periportal layers, mid-lobule layers, and pericentral layers in the liver, where purple curves link identical genes. (B) Heatmap of enriched ontology terms across the DEG lists (FC > 0.25), colored by p-values. (C) Lipid gene expression heatmap in liver. (D) A cartoon illustrates the hepato-intestinal axis, showing lipid metabolism processes, with red for upregulated and blue for downregulated gene expression in GF mice. (E) Schematic diagram of the ZBTB20-LPL axis regulating blood lipid levels in the liver. GR, glucose regulation; acyl-CoA, acyl-CoA metabolic; UFAS, unsaturated fatty acid synthesis; FAE, fatty acid elongase; TAGS, triacylglycerol synthesis; TAGH, triacylglycerol hydrolysis; CS, cholesterol synthesis; CT, cholesterol transport; FAO, fatty acid oxidation; LP, lipolysis; BAS, bile acid synthesis; LIP/R, lipoprotein and receptor; CM, chylomicron; SR- BI, scavenger receptor class B type I; CA, cholic acid; CDCA, chenodeoxycholic acid; CV, central vein; PV, portal vein; FA, fatty acid; ER, endoplasmic reticulum; VLDL, very low-density lipoprotein; IDL, intermediate-density lipoprotein; LDL, low-density lipoprotein.

**Figure S15** **Bile acid tissue distribution and hepatic zonation of metabolism-related genes in GF and SPF mice.** (A) Heatmaps illustrating the content of different bile acids among each sample. (B) Boxplots showing the ratios of CDCA to CA and β-MCA to CA in the cecum and feces of GF and SPF mice. Each point represents a sample, while horizontal lines depict the median, and the boxes indicate the 25th–75th percentile values. Differences between groups were analyzed using the Wilcoxon test. * *p* < 0.05, ** *p* < 0.001. (C) Expression variation of bile acid metabolism-related genes across eight hepatic zones between GF and SPF. CA, cholic acid; NorCA, norcholic acid; APCA, apocholic acid; CDCA, chenodeoxycholic acid; MCA, muricholic acid; HCA, hyocholic acid; UCA, ursocholic acid; UDCA, ursodeoxycholic acid; DCA, deoxycholic acid; NorDCA, nordeoxycholic acid; LCA, lithocholic acid; 3-oxoCA, 3-Dehydrocholic_acid; Dio-LCA, 7,12-Diketolithocholic acid; Iso-LCA, Isoallolithocholic acid; HDCA, hyodeoxycholic acid; 7-KetoLCA,7-ketolithocholic acid; T, tauro; G, glyco.


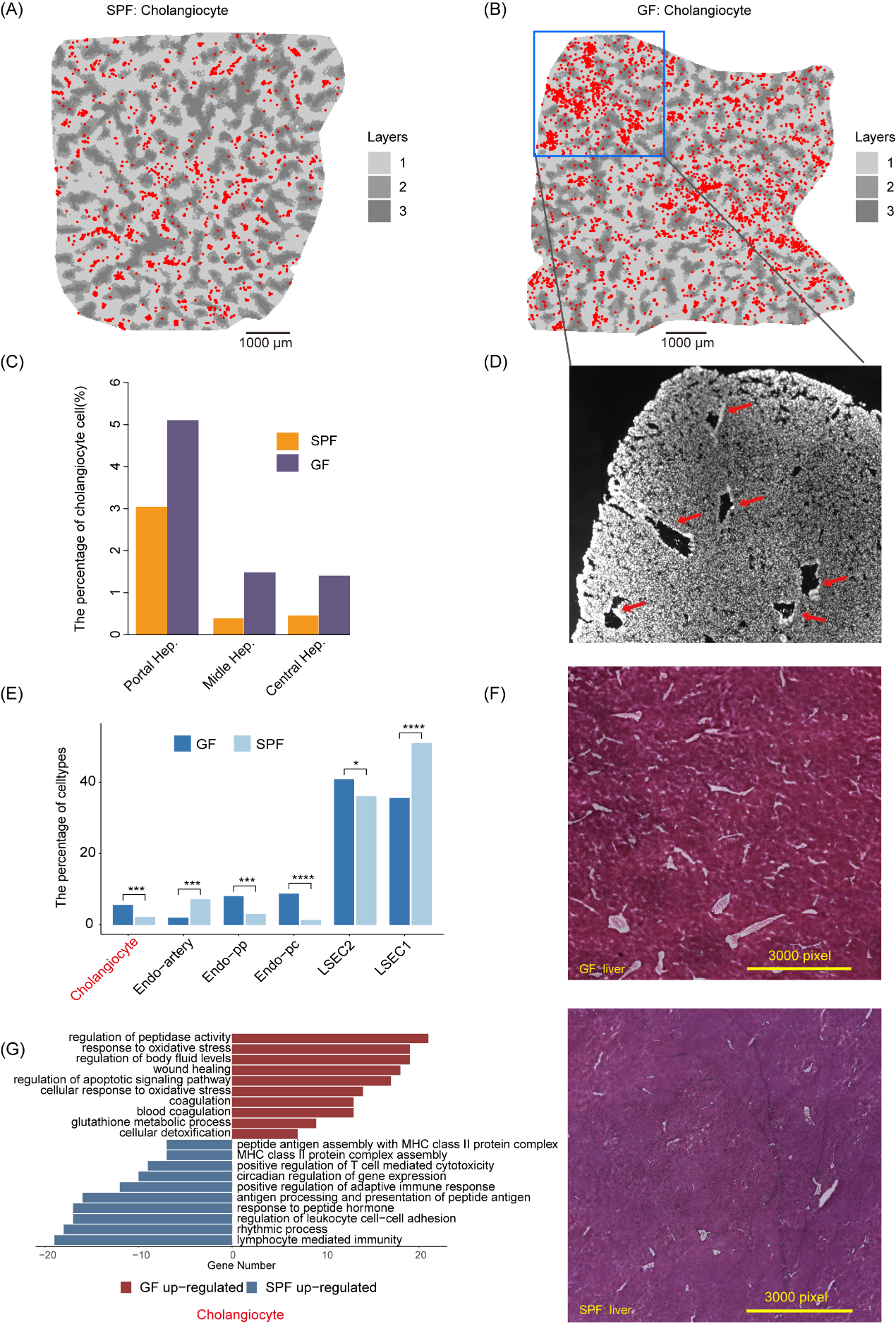


**Figure S16** Integrated analysis revealing perturbed bile ducts in GF mice. (A-B) Spatial visualization of cholangiocytes in the liver of SPF and GF mice. (C) The distribution of bile ducts in different zones of the hepatic lobule showed that GF and SPF bile ducts were concentrated around the portal vein. (D) Local magnification of ssDNA in the spatial transcriptome of the GF mice; the arrow points to the bile duct. (E) Proportion of different subtypes of endothelial cells in the liver of the GF and SPF mice. ns *p* > 0.05, * 1.0E-10 < *p* < 0.05, ** *p* ≤ 0.01, ** 1.0E-25 < *p* < 1.0E-10, *** 1.0E-50 < *p* < 1.0E-25, **** *p* < 1.0E-50. (F) HE staining of the liver of SPF and GF mice. (G) GO enrichment analysis of DEGs in the liver of GF (red) and SPF (blue) mice in cholangiocytes using scRNA-seq data. LSEC, liver sinusoidal endothelial cells; Endo-artery, artery endothelial cells; Endo-pp, periportal endothelial cells; Endo-pc, pericentral endothelial cells.
